# An ERP study on L2 syntax processing: When do learners fail?

**DOI:** 10.3389/fpsyg.2014.01072

**Published:** 2014-09-25

**Authors:** Nienke Meulman, Laurie A. Stowe, Simone A. Sprenger, Moniek Bresser, Monika S. Schmid

**Affiliations:** Center for Language and Cognition, University of GroningenGroningen, Netherlands; Research School of Behavioral and Cognitive Neurosciences, University of GroningenGroningen, Netherlands; Department of Language and Linguistics, University of EssexColchester, UK

**Keywords:** second language acquisition, grammatical gender agreement, event-related potentials (ERPs), P600, modality, immersion

## Abstract

Event-related brain potentials (ERPs) can reveal online processing differences between native speakers and second language (L2) learners during language comprehension. Using the P600 as a measure of native-likeness, we investigated processing of grammatical gender agreement in highly proficient immersed Romance L2 learners of Dutch. We demonstrate that these late learners consistently fail to show native-like sensitivity to gender violations. This appears to be due to a combination of differences from the gender marking in their L1 and the relatively opaque Dutch gender system. We find that L2 use predicts the effect magnitude of non-finite verb violations, a relatively regular and transparent construction, but not that of gender agreement violations. There were no effects of age of acquisition, length of residence, proficiency or offline gender knowledge. Additionally, a within-subject comparison of stimulus modalities (written vs. auditory) shows that immersed learners may show some of the effects only in the auditory modality; in non-finite verb violations, an early native-like N400 was only present for auditory stimuli. However, modality failed to influence the response to gender. Taken together, the results confirm the persistent problems of Romance learners of Dutch with online gender processing and show that they cannot be overcome by reducing task demands related to the modality of stimulus presentation.

## Introduction

Second language (L2) acquisition of many aspects of syntactic structure is known to be difficult, especially when acquisition starts later in life. A major question being debated in the literature is to what extent and under what circumstances late L2 speakers can become native-like with respect to syntax processing (e.g., Clahsen and Felser, 2006; White, 2007). The evidence is mixed; in some cases this does seem to be possible, while in other cases, it is difficult or impossible. A number of factors have been suggested to play a role in this variation, but two which have received relatively little attention are the difficulty of the target grammatical system and the potential role of modality of testing (written vs. auditory presentation). The present study investigates whether event-related potential (ERP) measures of native-likeness used in this line of research might be partially dependent on stimulus modality, as this might explain some of the inconsistency in the literature.

A structure that has frequently been used to test native-like attainment in the L2, is grammatical gender, since it has been shown to pose a major challenge to L2 learners (e.g., Hawkins, 2001; White et al., 2001; Sabourin, 2003; Blom et al., 2008). Demonstrating gender processing that is comparable to that of natives therefore forms a strong test for L2 syntax acquisition. Grammatical gender is a classification system for nouns (e.g., masculine and feminine in French, or masculine, feminine and neuter in German) which allows speakers to establish syntactic cohesion between the elements in a phrase through agreement. Because the gender of a word is typically not predictable from its meaning, learning grammatical gender involves acquiring both the knowledge of a word's gender (gender assignment) and of how gender is expressed syntactically (gender agreement or concord). Therefore, L2 learners must tag each new lemma with its corresponding gender and learn which grammatical elements in the context have to agree with it. For example in Dutch, all nouns are assigned to either the common or the neuter gender class and gender concord occurs with determiners and pre-nominal adjectives (e.g., *de_[def, common]_ tuin_[common]_*, the garden, *een_[indef]_ mooie_[indef, common]_ tuin_[common]_*, a beautiful garden). During processing, a comprehender must retrieve the noun's gender fast enough to establish gender concord. The question is (a) whether L2 learners manage to do so, and (b) whether they achieve this using the same processing strategies as native speakers.

Gender processing in L2 has already been the topic of numerous investigations using behavioral measures, such as grammaticality judgments, sentence-picture matching, (elicited) production, and eye tracking (for overviews, see, e.g., Grüter et al., 2012; Hopp, 2013). More recently, researchers have begun to employ ERPs to investigate native-likeness of grammatical gender processing in the L2, because ERPs are known to be highly sensitive to the immediate, unconscious on-line detection, and processing of linguistic anomalies (e.g., Osterhout and Holcomb, 1992; Molinaro et al., 2011). Studies using off-line behavioral measures (e.g., White et al., 2001, 2004; Franceschina, 2005) cannot give access to this sort of evidence, which makes interpretation of their results more difficult. Some online techniques such as eye tracking (Dussias, 2010) measure real-time language processing, but do not provide us with the qualitative evidence of potential brain mechanisms that ERPs can. The rationale of such ERP studies is that the more similar the response between native speakers and learners, the more similar the underlying neural and cognitive processing mechanisms. In other words, a comparison of ERPs in native speakers and L2 learners can tell us how native-like the latter really are.

In first language processing, gender and other (morpho)syntactic violations are found to be associated with two primary kinds of components: the left anterior negativity (LAN) and the P600. The LAN has been widely associated with morpho-syntactic agreement processes (Münte et al., 1993; Friederici et al., 2000; Molinaro et al., 2011), but others claim that it is a more general index of working memory load (Kluender and Kutas, 1993; Coulson et al., 1998). The P600 has been reported for a range of syntactic and other linguistic violations (e.g., Osterhout and Holcomb, 1992; Hagoort et al., 1993; Münte et al., 1993; Burkhardt, 2007). Given the extremely heterogeneous conditions that elicit a P600, this component cannot be exclusively associated with agreement specifically, or even syntactic processing difficulties more generally, and is therefore often interpreted as a late stage of (re)analysis of information (Osterhout and Holcomb, 1992; Bornkessel-Schlesewsky and Schlesewsky, 2008). It may even reflect a more general process, such as the P300 (Gunter et al., 1997; Coulson et al., 1998; but see Osterhout and Hagoort, 1998; Frisch et al., 2003). There is however, a strong correlation between the appearance of the P600 effect and grammatical violations. In contrast, findings are more varied with respect to the presence of a LAN. In addition to the LAN and P600, some studies have found an N400, or a biphasic N400-P600 pattern (but no LAN) in response to syntactic violations (see an overview reported in Molinaro et al., 2011). This is surprising, since the N400 is a component normally associated with difficulty in semantic integration (see Kutas and Federmeier, 2011, for an overview). It has therefore been proposed that an N400 in response to syntactic agreement anomalies is likely to be a result of non-syntactic information that is needed to process the mismatch, for example information that requires lexical access (Molinaro et al., 2011). Because the LAN and N400 are variable in studies of native processing, particularly for gender agreement, we will consider the P600 to be the primary measure of native-likeness, although we will report findings in the time window associated with the LAN/N400 (300–500 ms after presentation) as well.

ERP results regarding grammatical gender processing in the L2 have provided mixed results. A number of studies find that, at least under some conditions, sufficiently proficient L2 learners are able to show native-like ERP responses to gender violations. A set of studies investigating L2 processing of French suggests that English, German, and Spanish learners of French can show native-like ERP responses in the form of a P600 effect (Frenck-Mestre et al., 2009; Foucart and Frenck-Mestre, 2011, 2012). The same goes for English and Chinese learners of Spanish (Tokowicz and MacWhinney, 2005; Gillon Dowens et al., 2010, 2011). German and Polish learners of Dutch can also show a P600 in response to gender violations (Sabourin and Stowe, 2008; Loerts, 2012). Despite these consistent results, however, it is clear that this does not generalize to success in all aspects of gender processing, as the English and German learners also failed to respond in a native-like manner to gender in some forms of agreement (Foucart and Frenck-Mestre, 2011, 2012). Stronger yet, Romance learners of Dutch did not show sensitivity to gender agreement anomalies in the form of a P600 effect even in straightforward determiner noun agreement structures (Sabourin and Stowe, 2008). It is unclear why this group failed to exhibit the majority pattern; we will discuss some factors which might have affected their success in somewhat more detail.

One of the factors which has been considered to be central for native-like learning of a late L2 is whether a grammatical element (e.g., gender) is present in the L1. Many studies have focused on this question, but have reached different conclusions. There is some evidence that having a gender system in the L1 might be an advantage when acquiring an L2 gender system (e.g., Bruhn de Garavito and White, 2000; Hawkins, 2001; Franceschina, 2005). This is in favor of models proposing that the L1 restricts L2 acquisition (Hawkins and Chan, 1997). However, there is also evidence of L2 learners without gender systems in their L1 being able to show full acquisition of grammatical gender (White et al., 2001, 2004), which is seen as evidence against such a restriction (Schwartz and Sprouse, 1994, 1996; see also White, 1989; White et al., 2004). The presence vs. absence of gender in the L1 seems at the least to be more complicated than these views suggest, however.

The French and Spanish studies mentioned earlier show that learners with no gender in their L1 (English and Chinese speakers) can show native-like ERP responses. Further, Sabourin and Stowe (2008) find differences between two L1s which both have gender: German on the one hand and Romance learners on the other. Sabourin and Stowe themselves attribute their results to the (lack of) similarity between the native and target language of these learners: Dutch gender is in general predictable from the gender of the cognate German word due to their common historical origin, while there is no one-to-one-correspondence between Romance and Dutch gender at the lexical level. Moreover, agreement between noun and adjective is more similar in German and Dutch than the Romance languages and Dutch. Sabourin and Stowe conclude that processing routines are transferred from L1 to L2, rather than transfer of the abstract knowledge that nouns have gender, and that these routines must be similar for successful transfer (see Foucart and Frenck-Mestre, 2011, for a similar argument).

However, an explanation which assumes that similar routines in L1 are necessary for native-like processing does not account for the results of other studies mentioned above showing that even with no gender system in the L1, learners are able to show native-like effects. A different approach to the effects of L1 transfer is formulated within the Competition model (see Bates and MacWhinney, 1987). According to the competition-based account, when L1 does not contain gender there is no interference. This predicts successful outcomes for languages with no gender (Tokowicz and MacWhinney, 2005). However, when existing processing routines are transferred, they will cause interference if they are dissimilar from those required for L2 (accounting for the failure of the Romance learners of Dutch).

The target language itself may also contribute to the failure of Sabourin and Stowe's (2008) Romance group to show native-like processing. Most of the successful studies have investigated Romance target languages. Unlike Romance or Slavic languages, which have transparent gender systems (i.e., a predictable gender category based on morphophonological patterns), Dutch is generally regarded as having an opaque gender system (Corbett, 1991; van Berkum, 1996). Although some morphological forms predict the gender of the word, these cues are only available for a relatively small proportion of the vocabulary in the language. This clearly presents a more difficult problem for the learner than gender in a more transparent language, which may certainly explain why the Romance group in the Sabourin and Stowe study failed to achieve a native-like level.

Neither L1 interference nor target language opaqueness, however, entirely accounts for the results found by Loerts (2012). Her study demonstrates that highly advanced Polish learners of Dutch can show somewhat weak, but native-like ERP responses, even though Polish agreement differs from Dutch. Loerts' results also show that an opaque system can be learned, although it may be more difficult to learn than a transparent system. Only her most proficient learners showed native-like processing (see Davidson and Indefrey, 2009, for another example of relatively low proficient learners failing to show native-like effects for gender processing in an opaque L2 system), while even fairly low proficient English learners of Spanish have been shown to respond with a clear P600 effect (Tokowicz and MacWhinney, 2005). An alternative explanation is thus that Sabourin and Stowe's (2008) Romance learners were simply not proficient enough to show online processing comparable to that of natives. Although the proficiency of the Romance group was not investigated in detail, a similar group of German learners did significantly better when tested on offline gender knowledge (Sabourin, 2003). The Romance participants in the ERP study also performed worse at the end of sentence grammaticality judgments collected during the ERP session. It has been shown that proficiency affects brain responses (e.g., Steinhauer et al., 2006; McLaughlin et al., 2010). A replication of the Sabourin and Stowe study with a group of learners as proficient as in the Loerts study can demonstrate whether this is the sole explanatory factor. This is one of the aims of the current study.

However, there is another factor that may have produced the difference between the two Dutch studies, which has thus far been overlooked: testing modality. Unlike virtually all the other studies summarized above, Loerts (2012) tested her Polish learners using auditory sentence presentation. She argues that the learners had acquired their L2 primarily in the auditory modality as emigrants who arrived with no formal training in their new language. Consequently, processing routines may be tuned to the auditory stimulus modality. Indeed, the experience of learning in immersion can be expected to differ substantially from a formal learning environment. Yet, the various populations that have been tested so far differ in this domain. The participants in the Romance studies summarized above included learners with extensive formal training in their L2. In many of the studies there was no immersion (Tokowicz and MacWhinney, 2005; Gillon Dowens et al., 2011) or only minimal immersion during the participants' recent residence in France (Foucart and Frenck-Mestre, 2011, 2012). Sabourin and Stowe (2008), unlike Loerts, tested a similar late immersion population using visual materials, with each word presented consecutively in the center of the screen. An alternative explanation for the lack of a native-like response in their study could thus be difficulties with the visual presentation. Below, we will speculate about why a visual ERP paradigm might, under some circumstances, be problematic.

In a typical language comprehension ERP paradigm, participants are presented with sentences displayed one word at a time at the center of a screen, at a rate of around two words per second, a technique called rapid serial visual presentation (RSVP). The advantages of this method are that the duration of stimulus presentation can be controlled (and manipulated) tightly, that eye movements, which lead to large artifacts in the EEG, are reduced to a minimum, and that making the stimulus material and time-locking the brain responses to the presentation of violations in the stimulus is relatively straightforward. Consequently, a large majority of ERP sentence comprehension studies use this method. In contrast, auditory sentence presentation is used much less frequently in ERP research. With spoken stimuli, it is more difficult to control the presentation duration of individual words. In addition, making recordings of spoken sentences is more time consuming and requires tight control of acoustic confounds (e.g., prosodic cues about upcoming information, Dimitrova et al., 2012), as well as timing issues (e.g., setting markers to millisecond precision for the events of interest).

We do not expect to find interesting differences between word-by-word reading and listening for language processing in natives (Müller et al., 1997; Hagoort and Brown, 2000; Balconi and Pozzoli, 2005). In the L1, learners develop fully automatized processing of both modalities; moreover, the auditory representation of language is automatically activated by written materials (Perfetti et al., 1992; Frost, 1998), so that the routines activated during auditory processing can be utilized as well as those specific to the written modality (Homae et al., 2002). Despite expecting comparable results for the two modalities in general, even for L1 comprehenders, consecutive word by word presentation in the middle of the screen presents a challenge under some circumstances. The optimum speed of presentation is an issue; Hopp (2010) shows that speeded RVSP presentation can make even native speakers break down in their grammaticality judgment ability, making their performance mirror that of L2 learners (see also Camblin et al., 2007, who show a case where speeded RSVP eliminates an effect which is clear in naturally produced connected speech). Conversely, studies directed at optimizing computerized text presentation on small screens have shown that too slow a presentation can also interfere with comprehension (Bernard et al., 2001). This may result from working memory and maintenance issues. Stowe (1991) showed that readers were more likely to garden-path or have difficulty in recovering from a garden path with center of the screen presentation, as opposed to presentation of words across the screen in their normal position, even when readers were allowed to pick their optimum pace.

L2 learners differ in a number of ways from native speakers, some of which can be expected to interact with modality. First, their cumulative reading experience in the L2 is likely to be substantially lower than that of native speakers. This means that their activation of the L2 via this modality can be expected to be less automatized than in native speakers (Koda, 1996). Second, interference from the writing system of the first language may lead to even less activation of the phonological form of the L2, in comparison with natives (Koda, 1999). These differences can potentially play a role for all L2 learners, but may be especially relevant for learners with less formal instruction in the language and in whom learning took place primarily via the auditory modality. The optimum speed of presentation is also likely to differ between various groups of learners and natives. This issue has received relatively little attention in the literature, but given that stimulus modality was one difference between the unsuccessful Romance group reported by Sabourin and Stowe (2008) and the relatively more successful group studied by Loerts (2012), this factor was included in the current experiment in order to determine whether it explains the different patterns seen in the two studies. A clear effect of modality would suggest that researchers need to pay more attention to this variable in their experimental designs, and might have implications for the differences between immersed and instructed learners as well.

Summarizing, the goal of the current study is to gain more insight into why some groups may show persistent problems in attaining native-like processing of grammatical gender. We investigate grammatical processing in immersed Romance L2 learners of Dutch, using the P600 as a measure of native-likeness, in order to answer the question whether late L2 learners can show native-like syntactic processing, even if the gender marking in the L1 differs from that in the L2, which may cause interference, and the L2 gender system is relatively opaque, making it harder to recognize the grammatical agreement regularities. Following Sabourin and Stowe (2008), in addition to gender violations, which have proven difficult to master, we present our participants with non-finite verb violations, a construction that is relatively easy to acquire, as a baseline for comparison. We compare the responses of high-proficient Romance learners with those of native speakers of Dutch. Additional measures of proficiency will be gathered from the first. A within-subject comparison of stimulus modalities allows us to determine whether the absence of a P600 effect for gender in the Sabourin and Stowe (2008) study was due to processing demands associated with the task modality.

In addition to standard group analyses of the ERP waveforms, we will closely inspect individual differences within each group. Adding these analyses has several benefits. First, lack of effects in grand mean ERP results does not necessarily mean that none of the individuals showed a native-like ERP response. Rather, a null effect might be based on opposite effects (a positive going effect in one set of individuals and a negative going effect in others) canceling each other out. In a similar way, biphasic responses can be a spurious result of averaging (Osterhout, 1997; Nieuwland and Van Berkum, 2008; Tanner and Van Hell, 2014; Tanner et al., 2014). Before we draw any strong conclusion that a group of learners' processing of gender agreement qualitatively differs from natives, it is important to identify varying patterns in each of the groups. Furthermore, there may be predictors of native-likeness in L2 learners, such as age of acquisition, proficiency, language exposure and use, that may explain variance within the group (e.g., Weber-Fox and Neville, 1996, 1999; Rossi et al., 2006; Steinhauer et al., 2009; Tanner et al., 2014). Understanding which individual difference factors, if any, are associated with the outcome in L2 learning is a fundamental question which is difficult to answer with group-based analyses, and might also help us determine the source of some of the mixed patterns of results in L2 gender research.

## Materials and methods

### Participants

Participant characteristics and proficiency scores can be found in Table 1. Forty-five participants took part in the experiment. Seven participants had to be excluded from the analyses because of too many artifacts in the EEG signal. Nineteen of the remaining participants were Romance learners of Dutch (six French, five Italians, three Romanians, five Spanish). The remaining 19 participants were native speakers of Dutch. All participants were right handed, neurologically unimpaired and did not have any problems with hearing, speaking, or writing. Prior to conducting any procedures, written consent was obtained from all participants for the study, which was approved by the local ethics committee. Participants were fully debriefed at the end of the experiment and received a small fee for participation.

**Table 1:** **Means (and ranges) of participant characteristics and scores on proficiency measures, and significance of between-group comparisons (Mann-Whitney *U*-test)**.

**Measure**	**Learners (*n* = 19)**	**Natives (*n* = 19)**	***U*- and *p*-value**
**AGE/EXPOSURE/USE**			
Age at testing (years)	42.3 (24–64)	39.8 (21–59)	*U* = 162, *p* = 0.599
Age of acquisition (years)	26.0 (16–39)	–	–
Length of residence (years)	16.3 (5–43)	–	–
L2 use (%)^a^	58.4 (12.3–87.3)	–	–
**USE OF MODALITY: DURING LEARNING (%)^b^**			
Visual	43.7 (20–70)	–	–
Auditory	56.3 (30–80)	–	–
**USE OF MODALITY: CURRENT (%)^c^**			
Visual	42.6 (20–70)	–	–
Auditory	57.4 (30–80)	–	–
**PROFICIENCY MEASURES**			
C-test (%)^d^	79.4 (42.1–100)	95.2 (68.4–100)	*U* = 299.5, *p* < 0.001
Gender assignment task (%)^e^	87.3 (64.6–100)	99.5 (93.8–100)	*U* = 332.5, *p* < 0.001
**SELF-RATED PROFICIENCY^f^**			
Reading	4.4 (3–5)	–	–
Writing	3.6 (1–5)	–	–
Speaking	3.9 (2–5)	–	–
Listening	4.3 (3–5)	–	–

a *Composite score based on language use inside and outside of the home and use of Dutch media*.

b *Percentage of L2 use in the visual modality (i.e., reading/writing) compared to the auditory modality (i.e., speaking/listening) during learning of Dutch at onset of immigration*.

c *Percentage of L2 use in the visual modality (i.e., reading/writing) compared to the auditory modality (i.e., speaking/listening) in everyday life at the time of testing*.

d *Percentage of correct responses on the C-test (spelling errors were not penalized)*.

e *Percentage of correct responses (i.e., a minimum of 2/3 instances of each item assigned correctly) on the gender assignment task*.

f *Ratings on a 5-point scale with five as highest level of skill in Dutch*.

All learners had moved to the Netherlands at or after the age of 16 and had been immersed in the L2 context for at least 5 years at the time of testing. The learners had very little to no exposure to Dutch before immigration. They were asked to indicate the frequency of use of Dutch in daily life: a composite score for L2 use was calculated based on questions about language use at home (with partner and children), outside of the home (at the workplace and other), and use of Dutch media. They additionally answered questions about their use of Dutch in a specific modality: they estimated the percentage of use of the L2 in the visual modality (i.e., reading/writing) compared to the auditory modality (i.e., speaking/listening), both during learning of Dutch at onset of immigration and during everyday life at the time of testing.

L2 proficiency was assessed by means of several (written) measures. A pre-selection on the basis of a pre-test in the form of 20 grammar items of the Dutch DIALANG Placement Test (adapted from http://www.lancaster.ac.uk/researchenterprise/dialang/about.html) ensured that all participants had a relatively high level of proficiency in Dutch. Participants had to complete at least 13 of the items correctly to be selected for participation. Another proficiency measure was taken in the lab, in the form of a C-test (constructed by Keijzer, 2007), which consisted of two texts containing gaps where parts of some words had been left out. The participants' task was to fill the gaps. After the EEG experiment, participants were also asked to complete an offline gender assignment task. This task was used to test the participants' knowledge of the grammatical gender of the critical nouns used in the EEG experiment. In addition to these measures, learners rated their L2 Dutch in terms of reading, writing, speaking, and listening proficiency on a Likert-scale between 1 (very bad) and 5 (very good). Participants' scores on the proficiency measures can be found in Table 1.

### Materials

The design and materials of the EEG experiment were largely based on work by Loerts (2012), who studied L2 gender and non-finite verb processing in natives and Slavic learners of Dutch. One hundred and forty-four experimental sentences were created (see Table 2 for examples, the full list of sentences can be found in the Supplementary Material, Data Sheet 1). Forty-eight of the sentences^1^ were used to test non-finite verb agreement. Half of these contained an infinitive and the other half a past participle verb. For their ungrammatical counterparts, these verbs were altered into their participial or infinitival form, respectively. The other 96 sentences were used to test grammatical gender agreement. In these sentences, the determiner either agreed in gender with the following noun or violated gender concord. Determiner and noun were either adjacent, or non-adjacent (with an adjective intervening between the determiner and noun). Only highly frequent Dutch target nouns and verbs were used (nouns: mean = 2.16, range = 0.78–3.08; verbs: mean = 2.46, range = 0.95–4.05, on log lemma frequency of occurrence per million taken from the CELEX corpus: Baayen et al., 1995). Finally, 122 well-formed filler sentences were included. These filler sentences were added to raise the overall proportion of correct sentences to about 3/4, making the task more similar to natural language processing.

**Table 2:** **Example materials of the EEG experiment**.

**Condition**	**Example sentences**	**Number of items per list**
Non-finite verb agreement	Ze heeft alleen haar beste vriendin uitgenodigd/^*^uitnodigen voor haar verjaardag. (She has only invited/^*^invite her best friend for her birthday.) Hij probeert me altijd aan het lachen te maken/^*^gemaakt door grapjes te vertellen. (He always tries to make/^*^made me laugh by telling yokes.)	12/12 visual, 12/12 auditory
Gender agreement	Vera plant rode rozen in de/^*^het tuin van haar ouders. (Vera is planting red roses in the_com_/^*^the_neu_ garden of her parents.) Het duurde uren voordat Jeroen het/^*^de nette pak van zijn broer had aangetrokken. (It took hours for Jeroen to put on the_neu_/^*^the_com_ fancy suit of his brother.)	24/24 visual, 24/24 auditory

*Critical targets, where the ERP was measured, are underlined*.

For the auditory part of the experiment, spoken forms of all sentences were recorded. Each sentence was read aloud by a female native speaker with a standard Dutch accent who was trained to produce correct and incorrect sentences with normal intonation. Despite training, acoustic confounds, such as subtle prosodic cues to the upcoming ungrammaticality remain possible (Dimitrova et al., 2012). To prevent any influence of such confounds, each sentence was presented in its original form or in a digitally spliced version, constructed by cross-splicing the original recordings of grammatical and violation sentences, cutting at the onset of the determiner for the gender condition, or the verb in the non-finite verb condition. Noise reduction and volume normalization were applied to all sound files.

A within-subject design was employed to test the effects of modality within the same group of subjects. Eight experimental lists were created using a Latin Square design, crossing the factors modality (visual, auditory), correctness (correct, incorrect), and splicing (spliced, unspliced), to ensure each participant was presented with only one version of each sentence and an equal number of each type. Each list was presented to two or three participants from each group, and each participant saw only one list.

### Procedure

Event-related potentials were recorded while participants listened to or read the sentences. After each sentence, the participant had to make a grammaticality judgment. Participants were comfortably seated in an electrically shielded and sound attenuated chamber. The sentences were presented using E-prime (Schneider et al., 2002a,b), which in addition recorded accuracy with respect to the grammaticality judgments. Visual stimuli were presented on a computer screen in front of the participants. Speakers were placed to the left and right side of the screen. Visual sentences were presented at a rate of two words per second: each word was presented for 250 ms, followed by 250 ms blank screen. Auditory sentences were presented at normal speech rate. Participants were asked to avoid moving any parts of their body and not to move their eyes or blink during sentence presentation. The experiment consisted of four blocks: either two visual blocks followed by two auditory blocks or the reverse. The duration of the breaks between blocks was determined by the participant. Altogether, the EEG experiment lasted about 1 h.

Subsequently, participants were asked to fill in the pen and paper C-test. Finally, they performed a gender assignment task on a computer. The target words of the EEG experiment were presented in randomized order, each item appearing three times. Participants were instructed to indicate, by a mouse click on either the common (“de”) or neuter (“het”) definite article, whether they thought the word had common or neuter gender in Dutch.

### EEG recording and analysis

**Figure 1:**
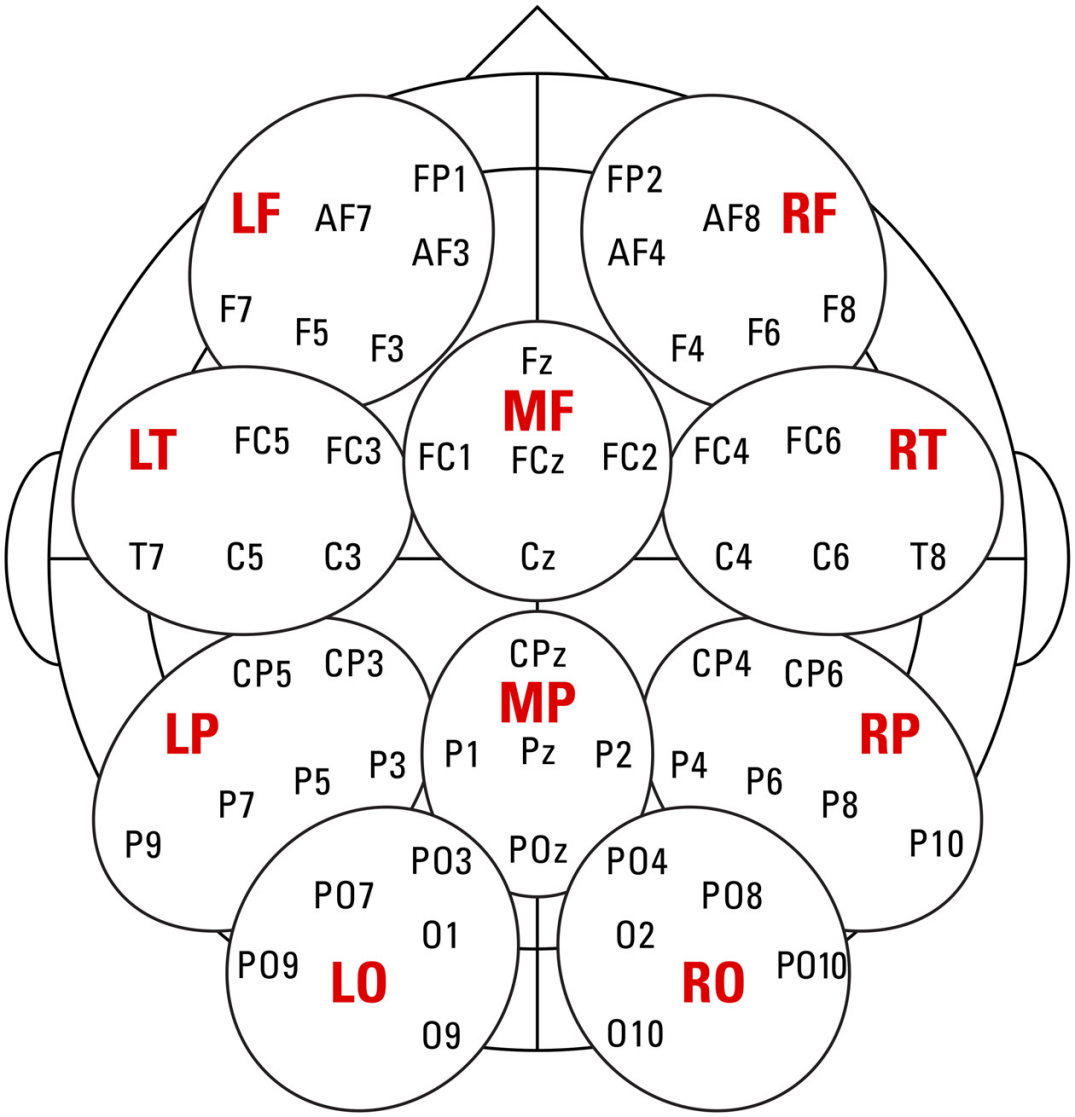
**Approximate location of the recording sites and the 10 regions of interest used for analyses: left/middle/right frontal (LF/MF/RF), left/right temporal (LT/RT), left/middle/right parietal (LP/MP/RP), and left/right occipital (LO/RO)**.

The continuous EEG (500 Hz/22 bit sampling rate) was recorded from 54 Ag/AgCl scalp electrodes mounted into an elastic cap (Electro Cap International, Inc.) according to the international extended 10–20 system (see Figure 1 for recording sites). To monitor eye-movements, four additional electrodes were placed on the outer canthi of each eye and above and below the left eye. Scalp electrode signals were measured against a common reference during recording. Impedances were reduced to below 10 kΩ ^2^. The amplifier (TMS international) measured DC with a digital FIR filter (cutoff frequency 130 Hz) to avoid aliasing. After acquisition, the raw data were further processed with Brain Vision Analyzer 2.0.4. The data were re-referenced to the average of two electrodes placed over the left and right mastoids and digitally filtered with a high-pass filter at 0.1 Hz and low-pass filter at 40 Hz. The data were segmented, time-locked to the onset of the critical target (from 500 ms before to 1400 ms after stimulus onset). Average ERPs were formed without regard to behavioral responses, from trials free of muscular and ocular artifacts; the latter were corrected using the Gratton and Coles procedure (1989). Individual channel artifacts led to rejection of 0.5% of the data in the learner group and 0.6% in the native group. A baseline period was set from 200 to 0 ms before onset of the critical words to normalize the data. A total of 10 regions of interest (ROIs), containing five or six electrodes each, were used for analyses (depicted in Figure 1).

We analyzed amplitudes of the ERP waveforms in the time-windows in which a LAN/N400 and P600 are to be expected: 300–500 and 600–1200 ms after stimulus onset. The latter window is somewhat longer than is typical in P600 studies in monolinguals, because the P600 in L2 learners can be somewhat delayed (Weber-Fox and Neville, 1996; Hahne, 2001; Rossi et al., 2006; Sabourin and Stowe, 2008). For grand mean analyses, ANOVAs were calculated within each time window and *sentence structure* (non-finite verb, grammatical gender) separately, using the ezANOVA function of the ez package (version 4.2.2: Lawrence, 2013), implemented in R (version 3.1.0: R Core Team, 2014). The analyses included *correctness* (grammatical, violation) and *modality* (visual, auditory) as within-participants factors, and *group* (natives, learners) as between-participants factor. Data from lateral (left and right frontal, temporal, parietal, and occipital ROIs) and medial (middle frontal and middle parietal ROIs) regions were treated separately in order to identify topographic and hemispheric differences. For the lateral regions, the ANOVA also included *hemisphere* (left, right) and *anterior-posterior* (frontal, temporal, parietal, occipital) as within participants factors. For the medial regions, *anterior-posterior* (frontal, parietal) was the only topographical factor in the ANOVA. The Greenhouse-Geisser correction was applied for violations of the sphericity assumption. Only main effects of, and interactions with, *correctness* are reported. In the presence of a significant higher-level interaction, lower-level interactions, and main effects are not interpreted. False discovery rate correction (Benjamini and Hochberg, 1995) was applied for follow-up tests to control for Type 1 error. Additional regression analyses, performed in R version 3.1.0 using the lm function of the lme4 package (version 1.1.6: Bates et al., 2014) will be described together with the results.

## Results

### Behavioral results

**Figure 2:**
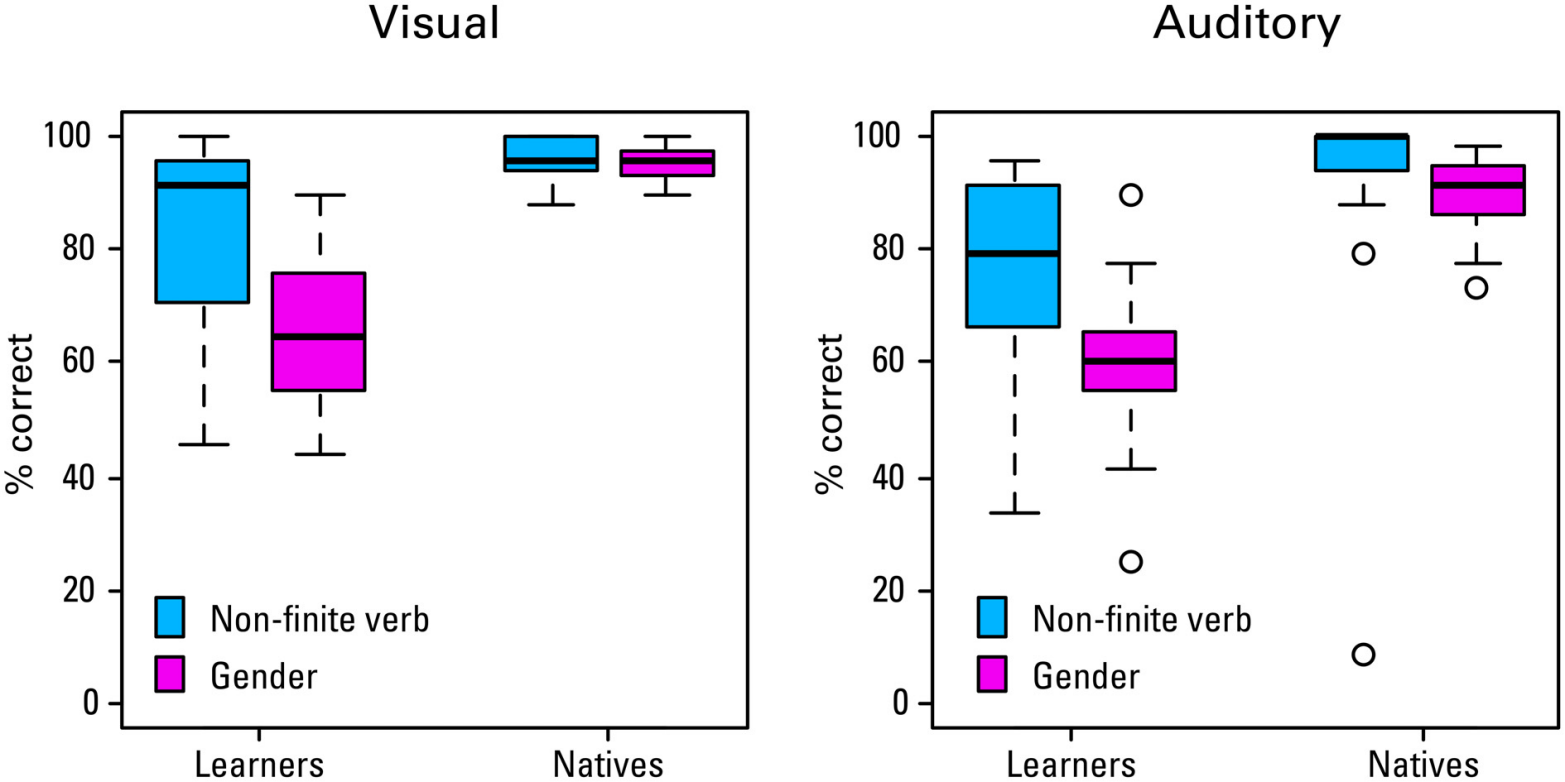
**Accuracy on grammaticality judgments made during ERP recording session by group, modality, and structure**.

The percentages of accurate grammaticality judgments per *group*, *sentence structure*, and *modality* are shown in Figure 2. A Three-Way ANOVA was conducted on the arcsine transformed proportions of correct responses to stabilizes variance and normalize the data (mean and SDs reported below are from the untransformed percentages). The ANOVA revealed a significant main effect of *group*, *F*_(1, 36)_ = 53.24, *p* < 0.001, with the learners giving fewer correct responses than the natives (mean = 71.1, *SD* = 17.8 vs. mean = 93.0, *SD* = 11.5). The main effect of *sentence structure*, *F*_(1, 36)_ = 41.66, *p* < 0.001, shows that the average performance is worse in the gender condition. However, there is also a significant interaction between *group* and *structure*, *F*_(1, 36)_ = 5.55, *p* = 0.024. Paired comparisons show that the difference between structures is highly significant in the learner group [*t*_(62.9)_ = 4.91, *p* < 0.001, gender mean = 62.8, *SD* = 14.1; non-finite verb mean = 79.5, *SD* = 17.3]. There is a smaller, but still significant difference between structures in the native group [*t*_(59.7)_ = 2.42, *p* = 0.019, gender mean = 92.2, *SD* = 6.2; verbs mean = 93.8, *SD* = 15.1]. Interestingly, with respect to one of our research questions, there is a significant main effect of *modality*, *F*_(1, 36)_ = 8.37, *p* = 0.006, with the percentage of correct responses in the auditory condition being somewhat lower than in the visual condition (mean = 79.5, *SD* = 20.0 vs. mean = 84.6, *SD* = 16.7). There are however no significant interactions between *modality* and *group*, *modality* and *structure*, or *group*, *modality*, and *structure* (all *F*s < 3).

### ERP results: grand mean analyses

**Figure 3:**
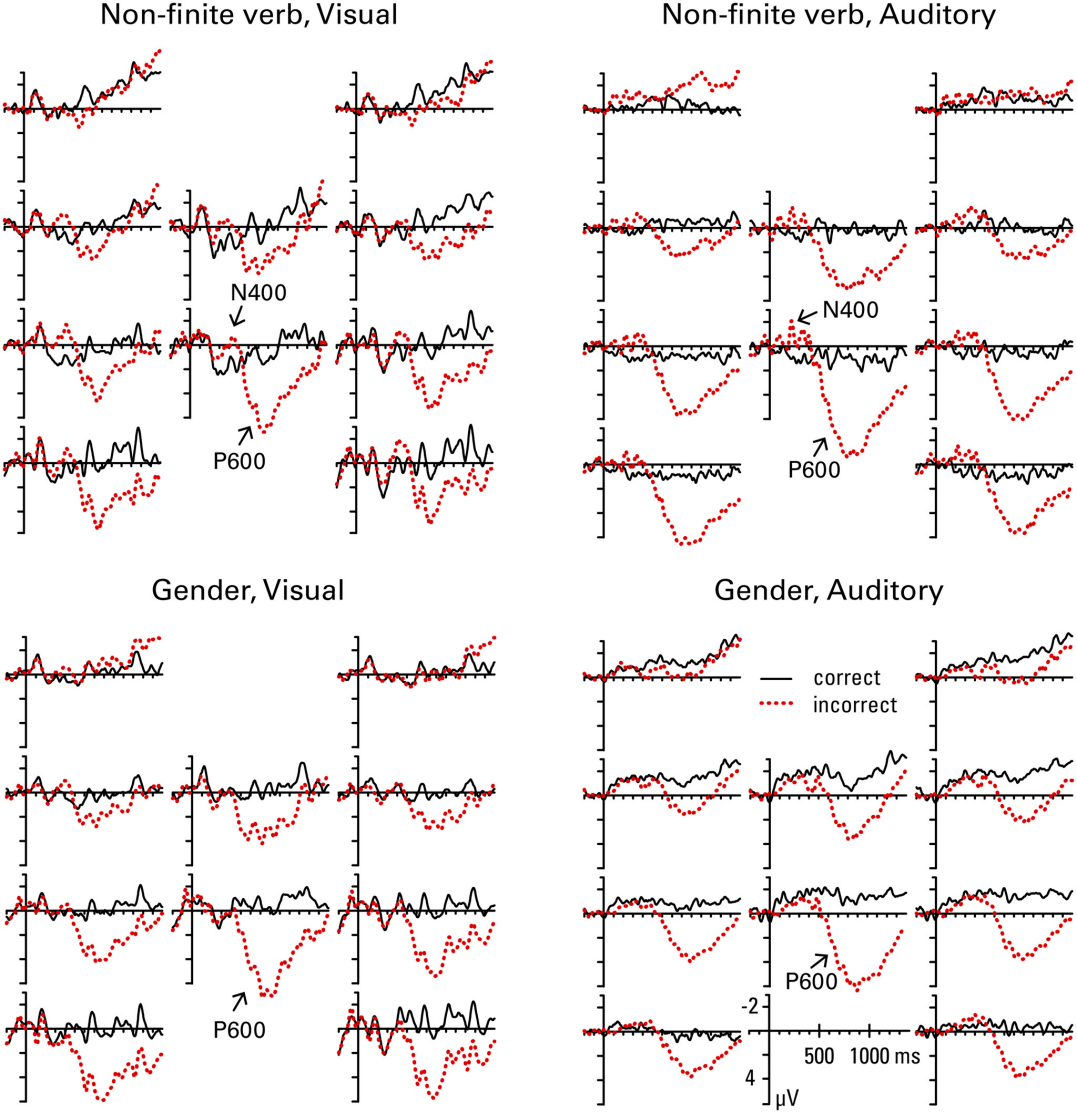
**Natives' grand average ERP waveforms at all 10 regions of interest (see Figure 1) for correct and incorrect use of non-finite verb and gender agreement in the visual and the auditory condition**.

**Figure 4:**
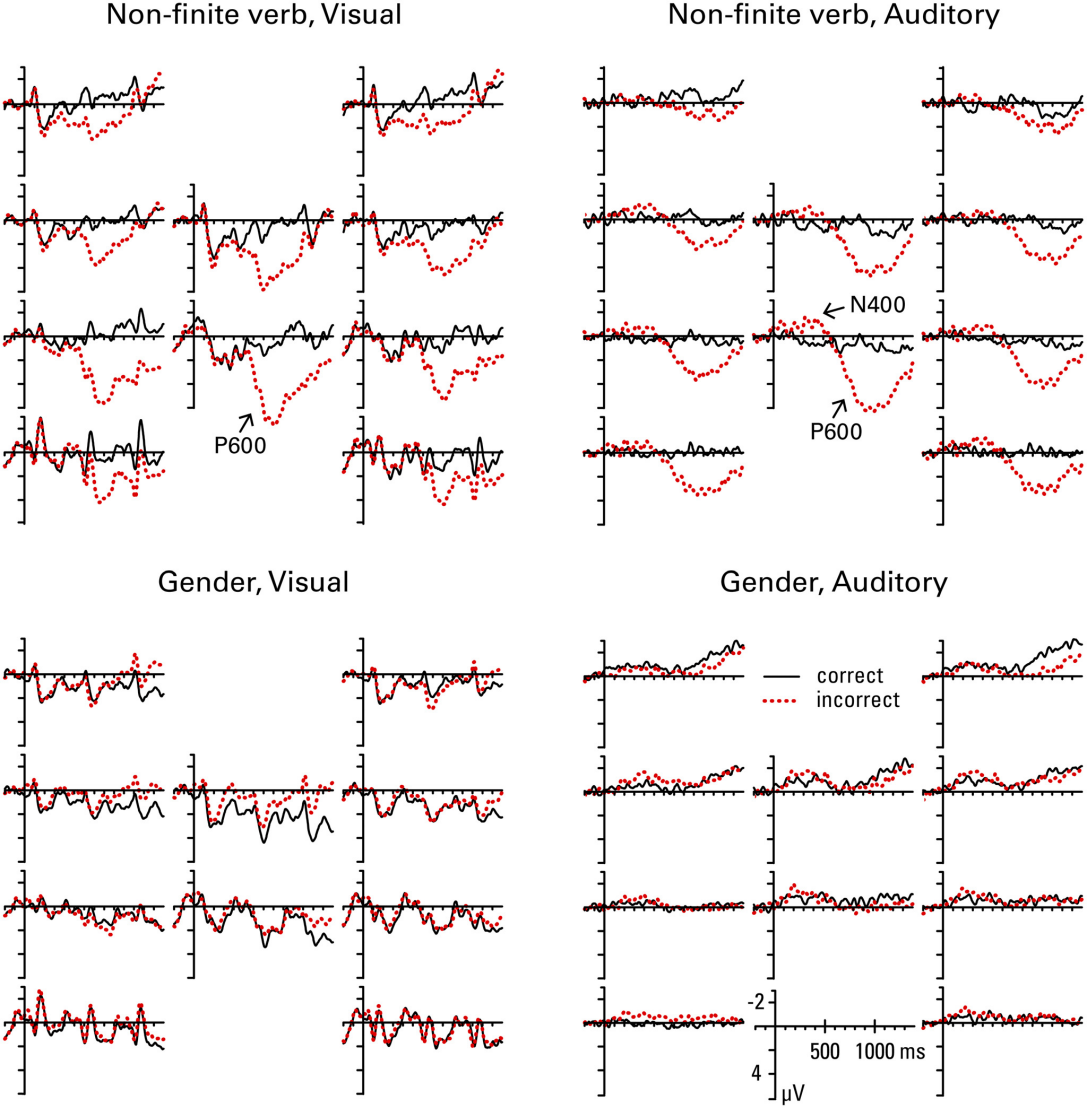
**Learners' grand average ERP waveforms at all 10 regions of interest (see Figure 1) for correct and incorrect use of non-finite verb and gender agreement in the visual and the auditory condition**.

Figures 3, 4 show the grand mean ERP waveforms for natives and learners, respectively. Results of the omnibus ANOVAs are provided in the Supplementary Material (Data sheet 2). Significant results and follow-up analyses will be described below.

#### Non-finite verb agreement

In the 300–500 ms window, the lateral omnibus ANOVA for the non-finite verb condition showed a significant *correctness* by *anterior-posterior* interaction, *F*_(3, 108)_ = 6.02, *p* = 0.011; follow-up analysis revealed that the effect of *correctness* reached significance in posterior regions only [frontal, *F*_(1, 36)_ = 0.52, *p* = 0.476; temporal, *F*_(1, 36)_ = 4.16, *p* = 0.065; parietal, *F*_(1, 36)_ = 14.70, *p* = 0.002; *F*_(1, 36)_ = 11.77, *p* = 0.004], with the incorrect condition showing more negative voltages than the correct condition. Due to a marginally significant *group* by *correctness* interaction in the omnibus ANOVA, *F*_(1, 36)_ = 3.65, *p* = 0.064, another follow-up analysis was conducted separately for natives and learners. This analysis revealed that the main effect of *correctness* was significant in natives, *F*_(1, 18)_ = 7.36, *p* = 0.028, but not in learners, *F*_(1, 18)_ = 0.08, *p* = 0.780. The medial omnibus ANOVA revealed a significant main effect of *correctness*, *F*_(1, 36)_ = 9.22, *p* = 0.004, with more negative voltages for the incorrect than the correct condition. Due to a marginally significant *correctness* by *anterior-posterior* interaction, *F*_(1, 36)_ = 3.33, *p* = 0.076, a follow-up analysis was conducted, which again revealed that the effect of correctness reached significance in the posterior region only [frontal, *F*_(1, 36)_ = 2.77, *p* = 0.105; parietal, *F*_(1, 36)_ = 14.55, *p* = 0.002]. Additionally, the omnibus ANOVA showed a marginally significant *group* by *correctness* by *modality* interaction, *F*_(1, 36)_ = 3.56, *p* = 0.067; but follow-up analyses failed to reveal a significant modality effect in either of the groups [*correctness* by *modality* interaction: natives, *F*_(1, 18)_ = 0.72, *p* = 0.407; learners, *F*_(1, 18)_ = 4.12, *p* = 0.114]. The main effect of *correctness* reached significance on its own in natives, *F*_(1, 18)_ = 6.26, *p* = 0.044, but not in learners, *F*_(1, 18)_ = 3.00, *p* = 0.100. Since visual inspection of the grand mean waveforms seems to suggest a possible negativity in medial regions for learners in the auditory condition, and finding a native-like effect in this time window for L2 learners is unusual, we performed an additional follow-up analysis separately for each modality in learners, which showed a significant *correctness* effect in the auditory, *F*_(1, 18)_ = 6.18, *p* = 0.046, but not the visual modality, *F*_(1, 18)_ = 0.43, *p* = 0.522.

In the later time window (600–1200 ms), the lateral omnibus ANOVA showed a significant *group* by *correctness* by *anterior-posterior* interaction, *F*_(3, 108)_ = 5.95, *p* = 0.008. Follow-up analysis revealed a significant main effect of *correctness* in both groups [natives, *F*_(1, 18)_ = 20.39, *p* = 0.001; learners, *F*_(1, 18)_ = 14.16, *p* = 0.001], with more positive amplitudes in the incorrect compared to the correct condition. A significant *correctness* by *anterior-posterior* interaction was present for natives only [natives, *F*_(3, 54)_ = 23.51, *p* = 0.001; learners, *F*_(3, 54)_ = 1.97, *p* = 0.169], which was driven by the fact that the positivity in natives was significant in the temporal, *F*_(1, 18)_ = 16.32, *p* = 0.001, parietal, *F*_(1, 18)_ = 36.07, *p* = 0.001, and occipital region, *F*_(1, 18)_ = 35.54, *p* = 0.001, but not the frontal region, *F*_(1, 18)_ = 0.00, *p* = 0.985. The medial omnibus ANOVA revealed a significant *correctness* by *anterior-posterior* interaction, *F*_(1, 36)_ = 22.93, *p* < 0.001; a follow-up analysis showed that the *correctness* effect is stronger in the parietal, *F*_(1, 36)_ = 68.36, *p* < 0.001, than the frontal region, *F*_(1, 36)_ = 29.15, *p* < 0.001.

It is apparent from these grand mean analyses that non-finite verb agreement violations are associated with a biphasic pattern of an N400 followed by a P600 in natives. The lack of significant effects for the frontal regions rules out a LAN effect in the 300–500 ms time window. Learners' responses are very similar to natives' in the later time-window (P600). However, in the early time window learners fail to show a native-like effect (N400) in the visual condition, and only show a smaller and less broadly distributed N400 compared to natives in the auditory condition.

#### Gender agreement

In the 300–500 ms window, the lateral omnibus ANOVA for the gender condition showed a significant *correctness* by *modality* by *anterior-posterior* interaction, *F*_(3, 108)_ = 3.90, *p* = 0.039, and a *group* by *correctness* by *modality* by *hemisphere* interaction, *F*_(1, 36)_ = 5.24, *p* = 0.028. Follow-up analyses conducted separately for natives and learners revealed a significant *correctness* by *modality* by *anterior-posterior* interaction in natives, *F*_(3, 54)_ = 6.28, *p* = 0.016, but no significant effects in learners (all *F*s < 2.03). However, in natives, neither the main effect of *correctness* nor the *correctness* by *anterior-posterior* interaction reached significance in either of the modalities analyzed separately (all *F*s < 3.90). The medial omnibus ANOVA showed a significant *group* by *correctness* interaction, *F*_(1, 36)_ = 4.30, *p* = 0.045. However, follow-up analyses failed to find a significant main effect of *correctness*, or any of its interactions, in either of the groups analyzed separately (all *F*s < 4.23).

In the 600–1200 ms window, the lateral omnibus ANOVA revealed a significant *group* by *correctness* by *anterior-posterior* interaction, *F*_(3, 108)_ = 20.17, *p* < 0.001, and a significant *correctness* by *modality* by *anterior-posterior* interaction, *F*_(3, 108)_ = 7.31, *p* = 0.002. Follow-up analyses conducted separately for natives and learners revealed a significant *correctness* by *modality* by *anterior-posterior* interaction in natives, *F*_(3, 54)_ = 6.17, *p* = 0.014, but no significant effects in learners (all *F*s < 1.81). In natives, the main effect of *correctness* was significant in all regions except for the frontal one [frontal, *F*_(1, 18)_ = 0.06, *p* = 0.806; temporal, *F*_(1, 18)_ = 14.33, *p* = 0.001; parietal, *F*_(1, 18)_ = 38.20, *p* = 0.001; occipital, *F*_(1, 18)_ = 35.39, *p* = 0.001], with amplitudes in the incorrect condition being more positive compared to the correct condition. The *correctness* by *modality* interaction did not reach significance in any of the regions (all *F*s < 4.03). The medial omnibus ANOVA showed a significant *group* by *correctness* by *anterior-posterior* interaction, *F*_(1, 36)_ = 11.24, *p* = 0.002. Follow-up analyses revealed that this was due to a significant *correctness* by *anterior-posterior* interaction in natives, *F*_(1, 18)_ = 26.82, *p* = 0.001, but not learners, *F*_(1, 18)_ = 1.86, *p* = 0.190. The interaction in natives was driven by the fact that the effect of *correctness* was stronger in the posterior region [frontal, *F*_(1, 18)_ = 13.04, *p* = 0.002; parietal, *F*_(1, 18)_ = 47.69, *p* < 0.001].

These grand mean analyses show that while natives show a classic P600 effect in response to gender agreement violations, learners do not: the P600 is absent for learners, in both modalities. In the early time window, there are again no effects for learners, while the natives seemed to show some small effects, which however failed to reach significance in follow-up analyses.

**Figure 5:**
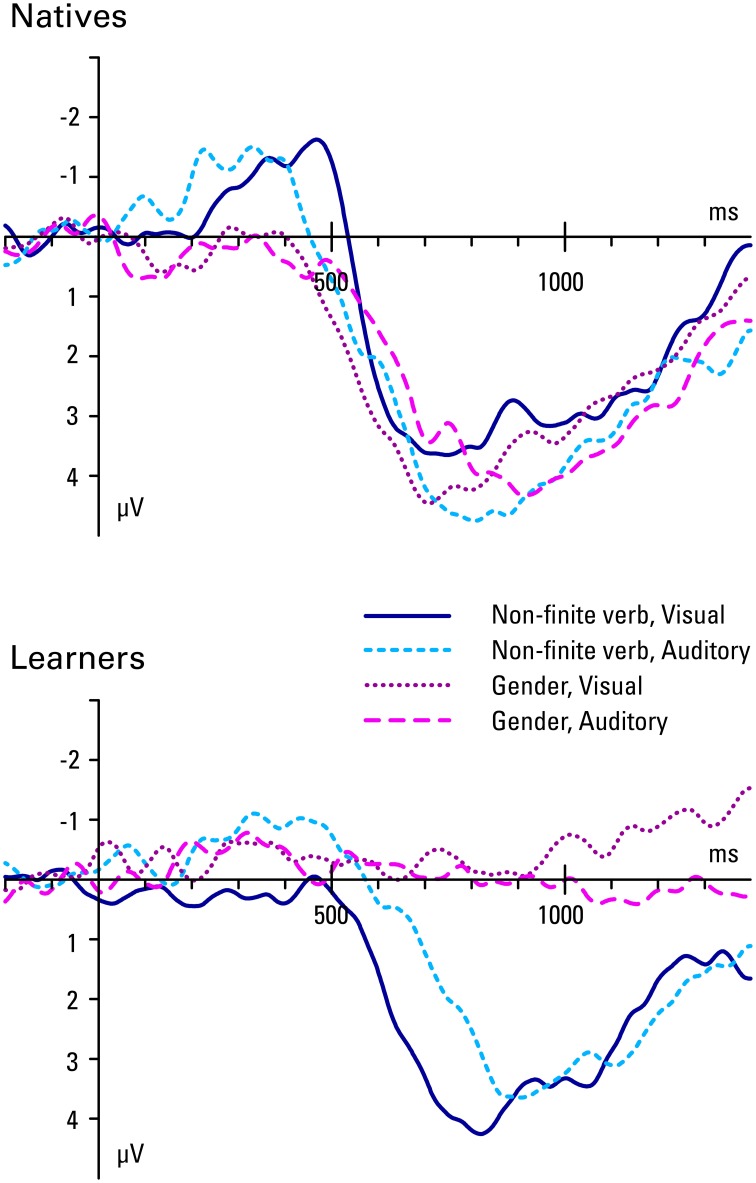
**ERP difference waves (incorrect minus correct sentence) per group, structure, and modality, collapsed over middle frontal and all temporal, parietal, and occipital ROIs**.

Figure 5 summarizes the P600 and N400 effects, showing the difference in amplitude between the violation condition and the grammatical condition, collapsed over middle frontal and all temporal, parietal and occipital ROIs, per group, structure, and modality. We see P600 effects for natives, preceded by an N400 effect in non-finite verb violations, but not gender violations. In contrast, the learners only show P600 effects for non-finite verb violations, but they do not show any effects of gender violation. The learners also show a small N400 effect for auditory non-finite verb violations (an effect that only reached significance in the medial regions).

### ERP results: individual differences analyses

In this section, we will have a closer look at individual differences. First, we will investigate the distribution of N400 and P600 effects across individuals, which can be of importance for the interpretation of the grand mean results, as discussed in the Introduction. Second, we will explore possible predictors of native-likeness in the learner group, since previous research has revealed that age of acquisition, length of residence, L2 proficiency and use can affect ERP responses (also discussed in the Introduction).

#### Closer inspection of the N400 and P600 patterns

Following work by Osterhout and colleagues (McLaughlin et al., 2010; Tanner et al., 2013, 2014) we regressed individuals' N400 effect magnitude onto their P600 effect magnitude, to investigate the distribution of these two components across individuals. The effect magnitude here refers to the average voltage difference between conditions: correct minus incorrect in the 300–500 ms window for the N400, and incorrect minus correct in the 600–1200 ms time window for the P600. Amplitudes were averaged across middle frontal and all temporal, parietal, and occipital regions, where the N400 and P600 effects are to be expected.

**Figure 6:**
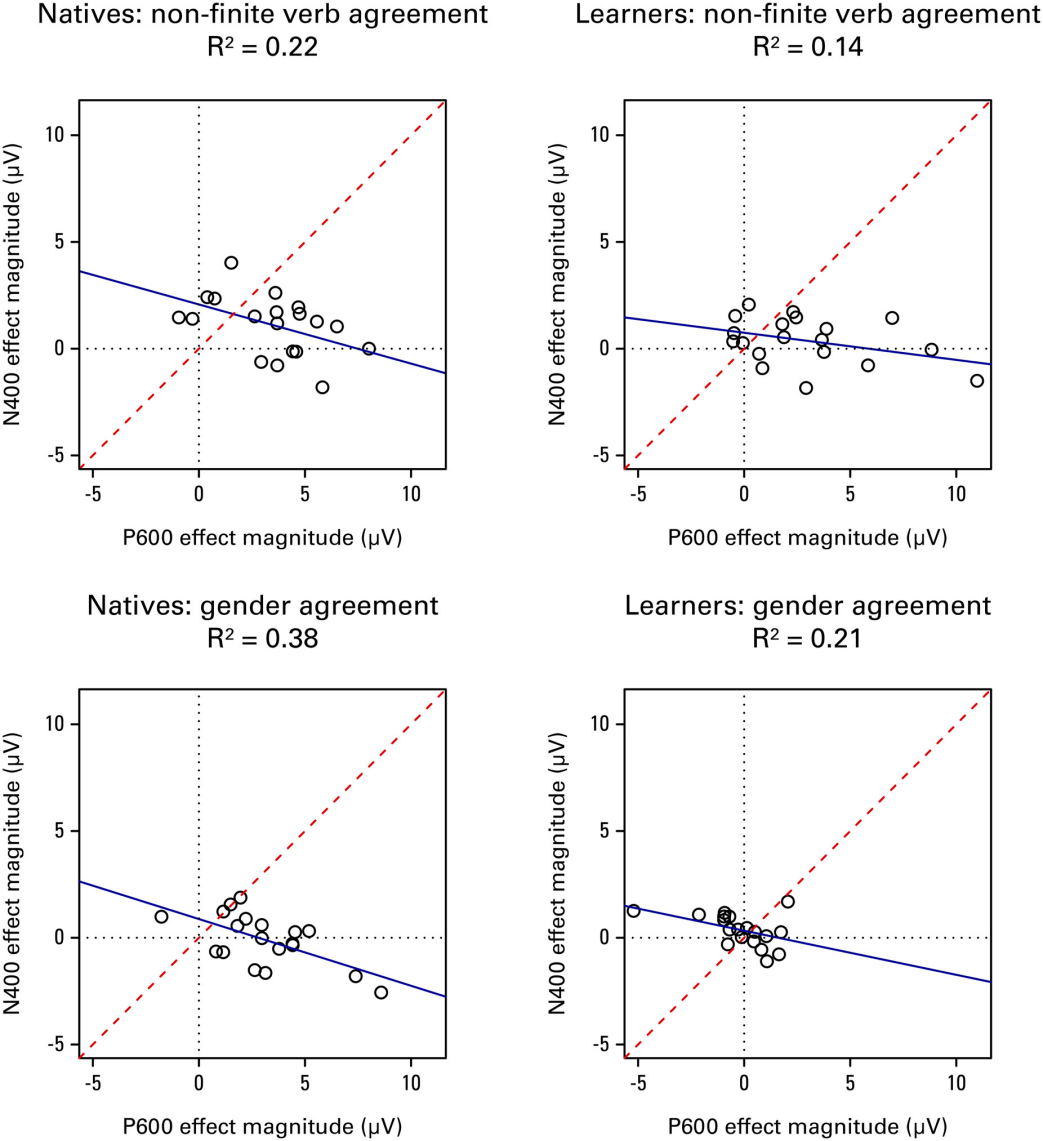
**The distribution of N400 and P600 effect magnitudes (correct minus incorrect for N400, incorrect minus correct for P600) across learners, averaged within middle frontal and all temporal, parietal, and occipital ROIs**. Each dot represents a data point from a single participant. The solid line shows the best-fit regression line. The dashed line represents equal N400 and P600 effect magnitudes: individuals above/to the left of the dashed line showed primarily an N400 effect, whereas individuals below/to the right of the dashed line showed primarily a P600 effect. In the non-finite verbs many individuals show biphasic responses (upper right quadrants), whereas in the gender condition there are more sustained positivities (lower right quadrants). Very few individuals show sustained negativities (upper left quadrants). Basically none of the learners are able to show sensitivity to gender violations.

Figure 6 shows the scatterplots of the results, for each group and sentence structure separately. We also investigated each modality separately, but since the results looked highly similar between modalities, these will not be discussed here. The figure informs us about whether the grand mean waveforms are representative of most individuals' ERP profiles. We concluded from our grand mean analyses that natives show a biphasic N400-P600 pattern for non-finite verb violations, and only a P600 for gender agreement violations. Examining Figure 6 we indeed see that the biphasic pattern is present for the majority of individuals in the non-finite verbs, and that a P600 (without preceding N400) is dominant for gender. The grand mean results of the learners showed native-like effects for verbs, but not for gender. This conclusion still holds if we look at individual patterns within the group: the distribution of responses in the verb condition looks highly similar between learners and natives, although there is a tendency toward more positivities without preceding negativities and less biphasic responses in the learners. The fact that basically none of the learners show any sensitivity to gender violations assures us that the null effect in the grand mean analysis was not due to a cancelation by different patterns.

#### Predictors of P600 effect magnitude in the learner group

To investigate which factors lead to a higher degree of native-likeness in L2 learners, we performed a multiple regression analysis (e.g., Baayen, 2008), to investigate the possible influence of age of acquisition, length of residence, L2 proficiency (as measured by the C-test), offline gender knowledge (as measured by the gender assignment task), and L2 use (composite score) on the P600. We took magnitude of the P600 as a measure of native-likeness, since the previous section revealed that this is the most reliable effect in the native group. The average amplitude of the difference wave (incorrect minus correct), calculated in the 600–1200 ms window collapsing middle frontal and all temporal, parietal, and occipital ROIs, was used as the dependent measure in the regression model. Because of skewed distributions, age of acquisition, and length of residence were log-transformed, and L2 proficiency, gender knowledge and L2 use were arcsine transformed prior to entry into the model. Additionally all predictor variables were centered at their mean. The correlation matrix for the dependent measure and the participant characteristics variables can be found in Table 3. Examining Table 3 we see that length of residence shows a significant positive correlation with gender knowledge (i.e., the ability to assign gender offline), *r*_(17)_ = 0.55, *p* = 0.014, with longer length of residence being associated with better gender knowledge. However, there is no relation between length of residence and the magnitude of the P600 (i.e., the ability to process grammatical structures efficiently online), *r*_(17)_ = −0.11, *p* = 0.665. L2 use positively correlates with both gender knowledge and P600 magnitude, *r*_(17)_ = 0.52, *p* = 0.023 and *r*_(17)_ = 0.49, *p* = 0.035, respectively, with a higher amount of L2 use being associated with better gender knowledge as well as larger P600 magnitudes.

**Table 3:** **Correlation matrix for the dependent measure and the participant characteristics variables used in the regression model**.

	**P600 magnitude**	**Log age of acquisition**	**Log length of acquisition**	**Arcsin of residence**	**Arcsin proficiency gender knowledge**	**Arcsin L2 use**
P600 magnitude	–					
Log age of acquisition	−0.083	–				
Log length of residence	−0.106	−0.147	–			
Arcsin proficiency	0.140	−0.327	0.230	–		
Arcsin gender knowledge	0.134	−0.416	0.552^*^	0.424	–	
Arcsin L2 use	0.486^*^	−0.388	0.413	0.293	0.518^*^	–

*Asterisk indicates significance of p < 0.05*.

**Figure 7:**
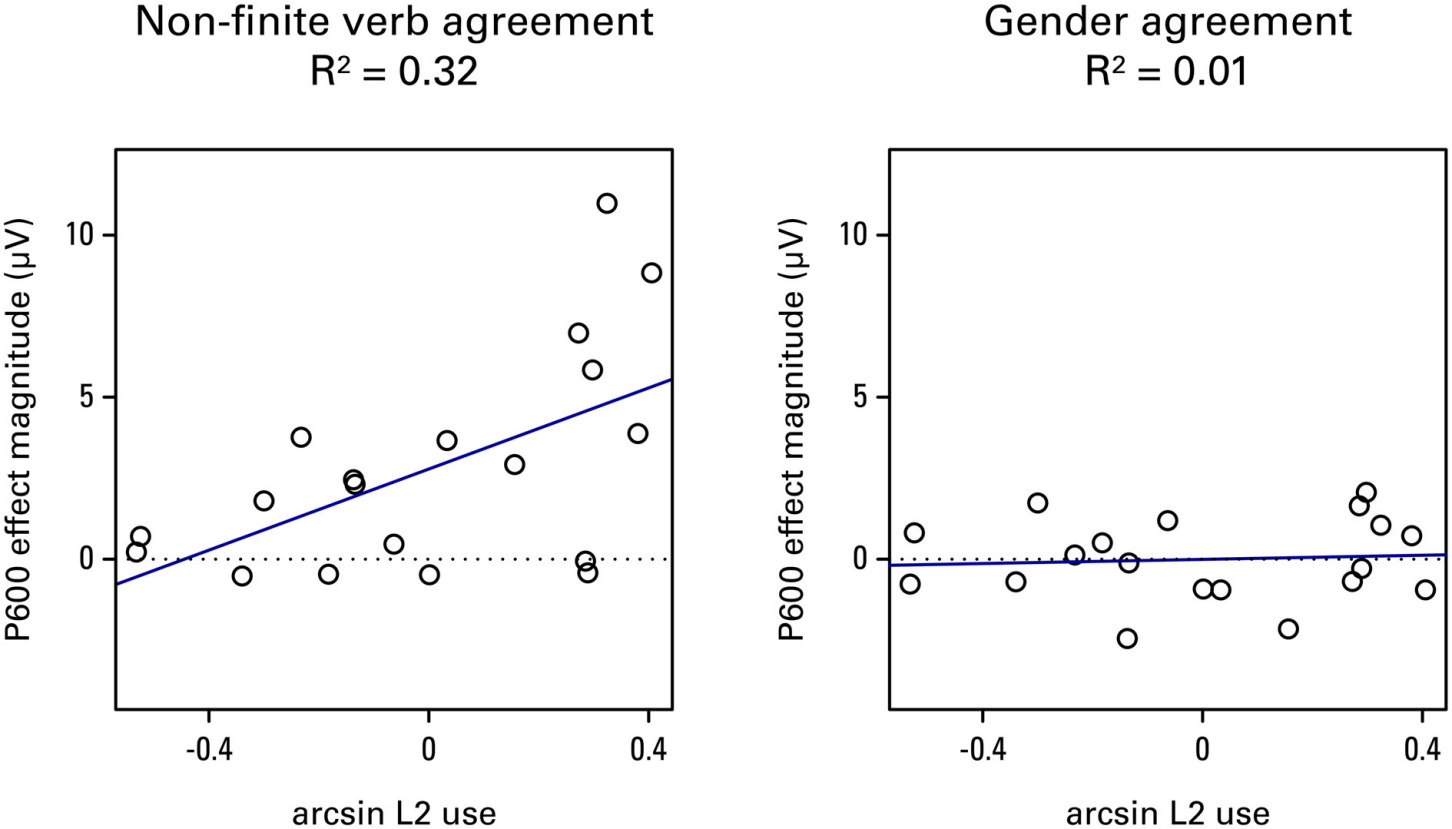
**The percentage of use of the L2 in daily life predicts P600 magnitude for non-finite verb agreement violations, but not gender agreement violations**.

In addition to the participant characteristics variables, structure and modality were tested as predictors in the model. The significance of predictors was evaluated by means of the *t*-test for the coefficients, in addition to model comparison using AIC (Akaike Information Criterion; Akaike, 1974). Table 4 shows the best linear multiple regression model (explained variance: 33.7%). This model shows that the structure being gender has a negative impact (β = −2.79, *t* = −4.41), and L2 use has a positive impact (β = 3.29, *t* = 3.07) on P600 effect magnitude. The other predictors (i.e., modality, age of acquisition, length of residence, proficiency, and gender knowledge) did not reach significance by themselves or in interaction with any other variables and were therefore not included in the model. Finally, the model additionally shows an interaction between the structure being gender and L2 use (β = −5.94, *t* = −2.78). This effect is plotted in Figure 7. There appears to be a significant effect of L2 use on the P600 for non-finite verb agreement violations, *R*^2^ = 0.32, *F*_(1, 17)_ = 8.08, *p* = 0.011, but no significant effect for gender agreement violations, *R*^2^ = 0.01, *F*_(1, 17)_ = 0.01, *p* = 0.756. No other significant interactions with structure or modality were found.

**Table 4:** **Linear multiple regression model predicting P600 effect magnitude in learners**.

**Predictor**	**Estimate**	***SE***	***t*-value**	***p*-value**
Intercept	1.388	0.316	4.390	<0.001
StructureIsGender	−2.789	0.632	−4.410	<0.001
L2use	3.288	1.070	3.074	0.003
StructureIsGender^*^L2use	−5.939	2.140	−2.776	0.007

## Discussion

Using the P600 as a measure of native-likeness, we tested whether sufficiently proficient late L2 learners can show native-like syntactic processing, even if (1) gender marking in the L1 is implemented differently and (2) the L2 gender system is opaque. We investigated the ERP responses of native speakers and Romance learners of Dutch to anomalies in constructions that are relatively easy to acquire (i.e., non-finite verbs) and those that have been shown to be more difficult (i.e., gender). In addition, we varied the modality in which the stimuli were presented, in order to investigate whether visual presentation might contribute to the lack of sensitivity to gender in the Romance group reported in previous research (Sabourin and Stowe, 2008). The non-finite verb violations elicited a biphasic N400-P600 effect in both native speakers and second language learners. However, in contrast to the native speakers, the learners only showed evidence of an N400 in the auditory and not the visual condition, although the statistical support for this difference is weak^3^. Also, the amplitude of the N400 effect was somewhat smaller than in the natives. For the gender violations, we found a clear P600 in natives, but not in L2 learners.

The effects of modality were quite subtle. We had hypothesized that increased processing demands in the visual modality might interfere with immersed learners' responses to grammatical violations and that they might show more native-like responses in the auditory modality. This hypothesis receives some support; the modulation of the N400 effect in non-finite verb violations in learners was in the hypothesized direction, with a native-like effect in the auditory but not the visual modality. However, for gender agreement learners failed to show sensitivity, regardless of the modality. Thus, the suggestion that the difference between Loerts' 2012) results for Polish speakers on the one hand, and Sabourin and Stowe's (2008) results and our current results for Romance speakers on the other, cannot be attributed to the difference in modalities.

In contrast to the modality effects, violation effects and group differences therein were robust. Before accepting the group patterns, it is important to examine the role of individual differences. A biphasic pattern may reflect the summation of single effects originating in two different groups of participants (Osterhout, 1997; Nieuwland and Van Berkum, 2008; Tanner and Van Hell, 2014; Tanner et al., 2014). Even more crucial for the current experiment, the absence of an effect in the L2 group may be due to variability, with some individuals showing the pattern found in native speakers, while others show no effect or even an opposing effect (Foucart and Frenck-Mestre, 2011). Inspection of individual differences for the gender violations confirmed that the grand average ERP patterns we report are representative of the majority of the individuals in each group. In contrast to natives, who consistently showed large P600 effects (Figure 6, bottom left panel), learners consistently failed to demonstrate any form of sensitivity to gender violations (Figure 6, bottom right panel). This result was confirmed by the fact that none of the participant characteristics we tested (increased proficiency or gender knowledge, earlier age of acquisition, longer length of residence or high percentage L2 use) was associated with a larger P600. In this sense, the current experiment replicates the pattern found by Sabourin and Stowe (2008); even highly proficient Romance learners of Dutch appear to have persistent difficulties in learning to use Dutch gender.

Turning to the non-finite verb violations, examination of the native speakers confirms that the biphasic pattern N400/P600 seen in this group is present in the majority of the individual participants (see Figure 6, top left panel). This biphasic effect in response to non-finite verb violations in natives has been found before (Kutas and Hillyard, 1983; Sabourin and Stowe, 2008; Loerts, 2012). As can be seen in Figure 6 (top right panel), many learners' responses were within the native range, showing evidence of the biphasic pattern, although this is primarily evident for the auditorily presented materials. Some individuals are less native-like; for this structure the P600 effect magnitude in the L2 group was found to be modulated by the percentage of use of the L2 in daily life. Use is not the only important factor for native-like attainment of syntax processing however; even the learners with the highest amount of daily practice in an immersed setting still show persistent problems with gender agreement.

Despite their failure to show native-like gender processing, the evidence suggests that the Romance learners are highly proficient. In addition to the off-line measures of proficiency (C-test and gender assignment) and online accuracy at ungrammaticality detection, which are within native range for a number of the participants, the evidence from the biphasic N400-P600 pattern provides a strong argument for high proficiency. Finding early ERP effects in response to grammatical violations like the N400 seen here is unusual in L2 research. Although both Loerts (2012) and Sabourin and Stowe (2008) found evidence of a biphasic pattern for their native groups, neither found the N400 in their L2 learner groups. According to Steinhauer et al. (2009), biphasic patterns are one of the latest stages of morpho-syntactic proficiency in late L2 acquisition. The fact that our learners were able to reach this stage for non-finite verb agreement, but that they cannot get past the initial stage of not showing any brain response differences for correct vs. incorrect use of gender agreement provides strong support for the difficulty of the acquisition of this element in Dutch L2 acquisition. This highlights the complexity of acquisition of the Dutch gender system, even by learners with a gender system in their L1. Furthermore, it emphasizes the fact that language learning aptitude is not an all or none phenomenon, but may vary widely between constructions.

Our results further illustrate the large discrepancy between online and offline processing measures in L2 acquisition research. Both the behavioral results of the gender assignment task and the sentence-final grammaticality judgments during the ERP recordings for gender violations indicate moderate to good knowledge of Dutch grammatical gender in the learner group. Yet, we observed a complete lack of response to these violations in the ERP signal. This reveals a discrepancy between offline knowledge of grammatical gender concord and the use of agreement knowledge during online processing. The lack of a significant relation between the magnitude of the P600 responses to gender violations and the score on the gender assignment task rules out the possibility that only learners with better offline performance are able to show online effects. The behavioral difference between the visual and the auditory modality, with performance being slightly worse for grammaticality judgments in the auditory modality, was also not reflected in the ERP signal for gender violations. These results illustrate that second language learners can develop successful strategies to cope with gender processing difficulties. These alternative routes, however, apparently take more time and are qualitatively different from what we observe in online native processing.

The results of the current study leave us with a puzzle; why do Romance learners of Dutch show such persistent problems with gender processing? Our results confirm that gender is difficult to process for late Romance learners of Dutch, compared with the results of studies targeting other languages. We replicated Sabourin and Stowe's (2008) findings, in the sense that our Romance learners likewise did not show native-like responses to gender violations, regardless of modality, although they showed responses to non-finite verbs that were close to the native model^4^. The factors most commonly suggested in the literature as to why gender or other forms of grammatical processing might be problematic do not appear to explain these results. Proficiency clearly plays some role in native-likeness in general (Steinhauer et al., 2006; McLaughlin et al., 2010), but as we argue above, our learners were quite proficient, certainly comparable to those in other studies in which learners have shown P600 effects for gender (Tokowicz and MacWhinney, 2005; Frenck-Mestre et al., 2009; Gillon Dowens et al., 2010, 2011; Foucart and Frenck-Mestre, 2011, 2012; Loerts, 2012). Also, our proficiency measure does not correlate with the magnitude of the ERPs.

Other potential explanatory factors involve the language experience of the learner, such as age of acquisition (Weber-Fox and Neville, 1996; Kotz et al., 2008) and exposure to and use of the L2 (Gardner et al., 1997; Flege et al., 1999; Dörnyei, 2005; Tanner et al., 2014). It is true that the studies reported by Frenck-Mestre and colleagues have generally tested earlier learners (with onset of acquisition in their teens rather than twenties and later). However, other studies have demonstrated native-like gender processing even for relatively late learners (Tokowicz and MacWhinney, 2005; Gillon Dowens et al., 2010). Furthermore, in the current study we did not even find a trend toward better performance for younger learners, making it again unlikely that this is the (only) decisive factor for native-likeness. The amount of L2 use also failed to explain the failure of the Romance learners to show online sensitivity to gender, even though, as our own results show, this can be important for native-likeness for other aspects of grammatical processing, like verb agreement. Length of residence, which impacts overall exposure, also showed no correlation with sensitivity to gender.

Failure to achieve native-like processing has also been linked to dissimilarity between L1 and L2 (Tokowicz and MacWhinney, 2005; Sabourin and Stowe, 2008; Foucart and Frenck-Mestre, 2011), as well as characteristics of the target language (Sabourin and Stowe, 2008; Loerts, 2012). Following this line of argumentation, Dutch and Romance languages may simply be too different from each other, which, combined with the fact that the Dutch gender system is relatively opaque, results in a very difficult challenge for native-like attainment. The lack of transparency of the Dutch gender system might explain why our Romance learners failed to show native-like processing for this characteristic of the language, as opposed to the much more transparent non-finite verb manipulation. For gender, previous research has shown that native-like processing is possible even in constructions with competition from an L1 gender system when a relatively transparent target gender system is to be acquired in L2 (Frenck-Mestre et al., 2009; Foucart and Frenck-Mestre, 2011; Gillon Dowens et al., 2011). In contrast, Loerts' study suggests that an opaque system is more difficult to acquire, since only her most proficient learners are able to show P600 effects, which are additionally somewhat smaller in amplitude compared to the natives. It remains an open question as to why, in contrast to Loerts (2012), even the most proficient learners in the current study did not show a P600. More research is needed to determine whether characteristics of the L1 or other (confounding) factors are at play in determining which individuals overcome the challenge of an opaque gender system.

One final point we would like to make is that, although we did not find extensive effects of stimulus modality, this factor is nevertheless of importance. As we noted, the early responses to ungrammaticality like the N400 in the biphasic response seen here are not generally found in late L2 learners, which has been taken as a sign of lack of native-likeness. It is possible that they have been missed due to the use of visual materials, since this effect was only seen in the auditory modality. Although we saw no effects on the amplitude of the P600 effect, certain populations may be affected more than others. Learners who do not share the same writing system in their L1 and L2, for instance, might have more difficulty automatizing their usage of the new alphabet (Koda, 1999; Wang et al., 2003). For these learners, the use of auditory materials might be a crucial prerequisite to obtain an accurate measure of their abilities. On the other hand, those whose learning has taken place with an emphasis on written materials may show less response when auditory materials are used. Given the large diversity of L2 speaker populations with respect to typological distance (both with respect to grammar and writing systems) and type of learning environment (immersion vs. classroom), it is important to be aware that the testing modality might influence the results, both in offline and online tests.

In conclusion, we can say that online grammatical gender processing is particularly difficult for Romance learners of Dutch, even at high levels of proficiency and with large amounts of L2 exposure and use in a natural setting, and regardless of testing modality. In contrast, responses highly similar to the native model are possible for a more regular and transparent structure (non-finite verbs), for which responses are modulated by both testing modality and L2 use. In contrast, the problems with gender are persistent and not affected by these factors, demonstrating the complexity of (late) L2 acquisition of the opaque Dutch gender system.

### Conflict of interest statement

The authors declare that the research was conducted in the absence of any commercial or financial relationships that could be construed as a potential conflict of interest.

## Footnotes

^1^Because of the large number of factors in the current design, it was not possible to get a high number of trials per condition without making the experiment too long, which in all probability would have resulted in severe fatigue effects in our data. We realize that as a result, the number of trials per condition is on the low side, particularly for the non-finite verb condition. However, highly salient agreement errors, such as the non-finite verb agreement violations used in the current study, have been shown to elicit large ERP effects. As the results section of this paper shows, even with this low number of trials we had sufficient power to find significant effects in this condition. In the less salient gender condition however, there was double the amount of trials per condition to ensure sufficient power.

^2^In some instances, some temporal and frontal electrodes could only be reduced to below 20 kΩ.

^3^We want to remind the reader that the modality effect in the non-finite verb condition should be interpreted with some caution. Unlike the main effects we report throughout the rest of the discussion (which are based on 24 and 48 items per condition for verb and gender, respectively), the marginally significant interaction we followed up on here is based on 12 items per condition only, which is relatively few for an ERP study. However, if we do not follow up on this interaction the main effect of correctness remains, suggesting that learners are like natives. We felt this claim would be too strong, and therefore discuss the follow-up analysis, despite the statistical concerns.

^4^Ones of the reviewers points out that having twice as many violation sentences in the gender condition than the non-finite verb condition, might be problematic, since less common stimulus types may elicit a P3 response (see Coulson et al., 1998; Hahne and Friederici, 1999). However, the difference waves shown overlaid in Figure 5 show that there is no difference in P600 effect magnitude between gender and non-finite verbs in the natives.
