# Supplementary material for: An ERP study on L2 syntax processing: When do learners fail?
Source: Front Psychol. 2014 Sep 25;5:1072. doi: 10.3389/fpsyg.2014.01072 (PMC4174886; doi:10.3389/fpsyg.2014.01072)
Supplement: Supplementary file 1 [file DataSheet1.PDF]

Supplementary Material

An ERP study on L2 syntax processing: When do learners fail?

Nienke Meulman[1,*], Laurie A. Stowe[1], Simone A. Sprenger[1], Moniek Bresser[2], Monika S. Schmid[1,3]

[1]Center for Language and Cognition, University of Groningen, Groningen, The Netherlands
[2]Research School of Behavioral and Cognitive Neurosciences, University of Groningen, Groningen, The Netherlands
[3]Department of Language and Linguistics, University of Essex, Colchester, United Kingdom

***Correspondence:**
Nienke Meulman
Center for Language and Cognition
University of Groningen
Oude Kijk in 't Jatstraat 26
P.O. Box 716, 9700 AS
Groningen, The Netherlands
n.meulman@rug.nl

Data sheet 1: Experimental Materials

First, the 48 sentences with non-finite verb agreement manipulations are shown, followed by the 96 sentences that were manipulated for grammatical gender agreement. The grammatical gender sentences contained an additional manipulation that is not discussed in the current study, namely cloze probability. Each gender sentence therefore has a high cloze (first sentence) and a low cloze (second sentence) version. Each participant was presented an equal amount of high and low cloze sentences per condition, and only one version of each sentence.

Non-finite verb agreement

Infinitive

1. Ik kan haar soms moeilijk begrijpen/*begrepen, omdat ze zo onduidelijk praat.
2. Omdat oma in Benidorm zat, wilde ze me niet bellen/*gebeld voor mijn verjaardag
3. Omdat we dit jaar geen vakantie in Zuid-Frankrijk kunnen betalen/*betaald, blijven we in Nederland.
4. Ik zag die mensen in het restaurant dansen/*gedanst op salsamuziek.
5. Nu hij moederziel alleen in New York zit moet hij veel denken/*gedacht aan zijn familie.
6. Joop moet op zaterdag zijn boodschappen doen/*gedaan voor de hele week.
7. Ik kan dit jaar helaas niet op vakantie gaan/*gegaan, omdat ik daar geen geld voor heb.
8. Wij willen dit jaar met de bus naar Spanje gaan/*gegaan om ons jubileum te vieren.
9. De mensen van het bureau willen hem advies geven/*gegeven over de verkoop van zijn huis.

10. Jongeren kunnen zich maar moeilijk voor politiek interesseren/*geïnteresseerd tegenwoordig.
11. Ondanks dat hij zijn biologische vader nooit heeft leren kennen/*gekend, weet hij toch veel over zijn leven.
12. We vonden het leuk om naar die voorstelling te kijken/*gekeken, maar het was jammer dat er geen pauze was.
13. Hij beloofde vóór acht uur te komen/*gekomen, maar om negen uur was hij er nog niet.
14. Na de operatie moest Kim nog een paar dagen in het ziekenhuis liggen/*gelegen ter observatie.
15. Hij probeert me altijd aan het lachen te maken/*gemaakt door grapjes te vertellen.
16. Zij kan deze puzzel oplossen/*opgelost binnen vijf minuten.
17. De NS adviseert niet per trein te reizen/*gereisd als dat niet strikt noodzakelijk is.
18. Ze houdt ervan lange brieven te schrijven/*geschreven op mooi briefpapier.
19. Linne zag haar vriend in de krant staan/*gestaan op een foto van Koninginnedag in Amsterdam.
20. Na zijn beroerte moest Pim zijn levensstijl aanzienlijk veranderen/*veranderd om herhaling te voorkomen.
21. Het vliegtuig naar Kuala Lumpur moest eigenlijk al om 20:55 uur vertrekken/*vertrokken van Gate E22.
22. Hij gaat een cursus volgen/*gevolgd aan de Universiteit van Utrecht.
23. Niemand kan zich de winter in Alaska voorstellen/*voorgesteld, want het is daar ongelooflijk koud.
24. Je moet te lang op de bus wachten/*gewacht in Groningen.

Past participle

25. Jouw les is om 11 uur begonnen/*beginnen in de zaal hiernaast.
26. Overal in de stad worden goedkope huizen gebouwd/*bouwen voor studenten en jongeren.
27. Ik heb hem nog nooit geloofd/*geloven toen hij zijn sterke verhalen vertelde.
28. Hij heeft het in Zuid-Spanje veel te warm gehad/*hebben en is blij dat hij weer in Nederland is.
29. De bevolking heeft twee jaar geleden een nieuwe regering gekozen/*kiezen en dat moet bijna weer gebeuren.
30. Pim is al twee keer te laat op zijn werk gekomen/*komen door een staking bij de spoorwegen.
31. Ik heb in maart een huis gekocht/*kopen aan de Amsterdamse gracht.
32. Ze hebben van de directeur een cadeautje gekregen/*krijgen voor de kerstdagen.
33. Ik heb die roman al gelezen/*lezen toen ik nog maar 12 jaar was.
34. Sofie heeft gisteren 5km gelopen/*lopen door de bossen in Drenthe.
35. Hij had zich plotseling omgedraaid/*omdraaien toen hij zijn naam hoorde.
36. Ik had mijn auto verkeerd geparkeerd/*parkeren en vond een fikse bekeuring onder de ruitenwisser.
37. Hij heeft uitsluitend over zijn verleden gepraat/*praten tijdens het interview.
38. De trein werd gisteren meteen gerepareerd/*repareren na de heftige botsing met een goederenwagon.
39. Hij heeft per ongeluk de thee naast het kopje geschonken/*schenken, maar hij ruimt het netjes weer op met een doekje.
40. De slager heeft zich in zijn vinger gesneden/*snijden met een scherp mes.
41. De directeur heeft de situatie aan de werknemers uitgelegd/*uitleggen tijdens de lunchpauze.
42. Ze heeft alleen haar beste vriendin uitgenodigd/*uitnodigen voor haar verjaardag.
43. Ik ben vroeger een keer in de sloot gevallen/*vallen met de fiets.
44. Die man heeft gisteren een groot ongeluk veroorzaakt/*veroorzaken vlakbij het treinstation.
45. We hebben de hele middag op hem gewacht/*wachten bij het gemeentehuis.

46. Die man heeft zijn hele leven hard gewerkt/*werken en gaat volgende maand met pensioen.
47. Ik heb in Utrecht gewoond/*wonen totdat ik 18 jaar was.
48. Ik ben vreselijk boos op hem geworden/*worden, omdat hij had gelogen over zijn minnares.

Grammatical gender

Determiner-noun, common gender

1. Dennis werd na te hard rijden bekeurd door de/*het agent met de boze blik.
   Dennis werd na het feestje gevolgd door de/*het agent met de boze blik.
2. De vader van Hans parkeerde de/*het auto op het parkeerterrein en deed een middagdutje.
   De vader van Hans zette de/*het auto op het parkeerterrein en deed een middagdutje.
3. Pieter ligt al de hele dag op de/*het bank televisie te kijken.
   Pieter heeft per ongeluk zijn sleutels op de/*het bank laten liggen.
4. Nadat hij de koeien had gemolken ging de/*het boer aan het werk op het land.
   Nadat hij een gezond ontbijt had gehad ging de/*het boer aan het werk op het land.
5. Sjoerd schrijft de/*het brief aan zijn verloofde die in Frankrijk woont.
   Sjoerd krijgt de/*het brief van zijn verloofde die in Frankrijk woont.
6. Op 1 mei viert de arbeidersbeweging jaarlijks de/*het dag van de arbeid.
   Elk jaar verheugen mensen zich wereldwijf op de/*het dag van de arbeid.
7. Kees heeft alweer een lekke band en leent vandaag de/*het fiets van zijn zus.
   Kees gaat vandaag vroeg naar zijn werk en neemt de/*het fiets van zijn zus mee.
8. De blinde vrouw durfde pas over te steken toen iemand haar bij de/*het hand nam bij het stoplicht.
   De aardige vrouw stond bij het stoplicht en nam de/*het hand van het jongetje dat over wilde steken.
9. Marianne wandelt dagelijks met de/*het hond door het park.
   Lisanne winkelt dagelijks met de/*het hond aan de riem.
10. Het muisje werd ’s avonds opgegeten door de/*het kat van de buren.
    Het meisje werd ’s avonds aangevallen door de/*het kat van de buren.
11. Jakob gaat iedere zondag bidden in de/*het kerk in zijn woonplaats.
    Jakob bakt verse koekjes voor de/*het kerk in zijn woonplaats.
12. Marieke heeft geen idee hoe laat het is en kijkt naar de/*het klok in de keuken.
    Marieke heeft speciaal voor haar moeder de/*het klok in de keuken goed gezet.
13. De docent vraagt om stilte aan het begin van de/*het les over de Middeleeuwen.
    Annette schilt een appel aan het begin van de/*het les over de Middeleeuwen.
14. Silvia gaf de huilende baby aan de/*het moeder en zette koffie.
    Silvia bracht verse koffie naar de/*het moeder van de tweeling.
15. De arts besloot het slechte nieuws persoonlijk aan de/*het patiënt te vertellen.
    De man besloot het slechte nieuws persoonlijk aan de/*het patiënt te vertellen.
16. De leerlingen gaven de docenten van de/*het school een groot cadeau als dank voor de leuke lessen.
    De burgemeester gaf de werknemers van de/*het school een groot cadeau als dank voor de goede resultaten.
17. De wandelaar struikelde over de/*het tak in het bos.
    De ambtenaar kocht de/*het tak voor in zijn vensterbank.
18. Onze nieuwe woning bevindt zich op de hoek van de/*het straat vlak bij het bos.
    Maurits en Willem hebben om acht uur afgesproken in de/*het straat bij het bos.

19. Jorien gaat op uitwisseling naar Spanje en zal daar een cursus volgen om de/*het taal te leren.
    Jorien verveelt zich nogal snel en heeft besloten dat ze de/*het taal van haar oma wil leren.
20. De gezonde jongen gaat nooit met de lift, maar neemt altijd de/*het trap naar de tweede verdieping.
    De gezonde jongen gaat vaak onderuit, maar neemt altijd de/*het trap naar de tweede verdieping.
21. Vera plant rode rozen in de/*het tuin van haar ouders.
    Vera breit een sjaal in de/*het tuin van haar ouders.
22. De student bezoekt veel monumenten tijdens de/*het vakantie op Ameland.
    De student leert nieuwe talen tijdens de/*het vakantie op Ameland.
23. Tijdens het debat beantwoordt de politica de/*het vraag over het milieu.
    Tijdens het feest kreeg de organisator de/*het vraag over het milieu.
24. Richard wil in tachtig dagen een reis om de/*het wereld maken.
    Richard wil na zijn studie de/*het wereld helpen te verbeteren.

Determiner-noun, neuter gender

25. Marijn kreeg een ontzettend moeilijke vraag van de leraar, maar hij wist het/*de antwoord wel meteen.
    Marijn kreeg het ontzettend warm en benauwd, want hij kon het/*de antwoord op de vraag niet geven.
26. Laurens slaapt aan de linkerkant van het/*de bed, omdat die kant lekkerder ligt.
    Laurens zit verveeld tv te kijken het/*de bed in zijn slaapkamer.
27. Steven is benoemd tot de nieuwe directeur van het/*de bedrijf van zijn vader.
    Steven bezoekt iedere vrijdagmiddag het/*de bedrijf van zijn vader.
28. De bakker in ons dorp bakte het/*de brood volgens speciaal recept.
    Mijn buurvrouw maakte het/*de brood volgens speciaal recept.
29. Peter zette de bos bloemen naast de computer op het/*de bureau van de zakenman.
    Peter vond de speelgoedauto naast de snoeppot op het/*de bureau van de zakenman.
30. Met een jacht voer het jonge stel naar het/*de eiland in de Zuidzee.
    Na het huwelijk gingen de ouders naar het/*de eiland om verder te feesten.
31. Als de drukke consultant om zes uur thuiskomt heeft zijn vrouw het/*de eten al klaar staan.
    Als de drukke consultant uit zijn vergadering komt heeft zijn secretaresse het/*de eten al klaar staan.
32. Johan betaalde de motor met het/*de geld dat hij voor zijn verjaardag kreeg.
    Johan was erg blij met het/*de geld dat hij voor zijn verjaardag kreeg.
33. De interviewer stelde zeer interessante vragen tijdens het/*de gesprek met de minister-president.
    De oliemagnaat stond uit verveling lange tijd te gapen tijdens het/*de gesprek met zijn werknemers.
34. Karel heeft zo'n ongelofelijke dorst dat hij in één keer het/*de glas water opdrinkt.
    Karel rent de keuken in en ziet daar het/*de glas water op een dienblad staan.
35. De cardioloog onderzocht met spoed het/*de hart van de zieke jongen.
    Tijdens de biologieles stond het/*de hart van de jongen in vuur en vlam.
36. Het gelukkige en pasgetrouwde stel ging samenwonen in het/*de huis aan het water.
    Aan de overkant van het meer zagen de puberale jongens het/*de huis aan het water.
37. Japan staat bekend als het/*de land van de moderne technologie.
    Jacob was niet bekend met het/*de land van de moderne technologie.
38. Het werd pikkedonker toen plotseling het/*de licht uitviel.
    Het werd gevaarlijk in de keuken toen plotseling het/*de licht uitviel.

39. In de winter schaatst Henk dagelijks op het/*de meer achter zijn huis.
    In de winter zit Henk graag bij het/*de meer achter zijn huis.
40. De journalist kijkt iedere ochtend naar het/*de nieuws op televisie.
    De pianist is altijd erg geïnteresseerd in het/*de nieuws op televisie.
41. Voor de inzamelactie bedacht Dorothea het/*de plan om een sponsorloop te houden.
    Het verslag bestond onder andere uit het/*de plan om een sponsorloop te houden.
42. Jeroen Pauw is bekend als presentator van het/*de programma Pauw & Witteman.
    Jeroen heeft een ontzettende hekel aan het/*de programma Pauw & Witteman.
43. Voor de speciale groentesaus volgde Arjan het/*de recept dat hij van zijn oma kreeg.
    Speciaal voor oudejaarsavond haalde Arjan het/*de recept van zijn oma van zolder.
44. De herder scheert ieder jaar de wol van het/*de schaap voor de verkoop.
    De handelaar controleert de kwaliteit van het/*de schaap voor de verkoop.
45. Haar fiets vergrendelde Natasja met het/*de slot gemaakt van staal.
    Naast het keukenblok vond Natasja het/*de slot gemaakt van staal.
46. De schipper legt een stevige knoop in het/*de touw van de mast en vertrekt dan richting Ameland.
    De schipper ziet plotseling het/*de touw van de mast losgaan en rent er direct naartoe.
47. De broers lijken veel op elkaar, maar als je goed kijkt zie je het/*de verschil tussen beide.
    De broers kijken samen naar het schilderij en zien dan het/*de verschil tussen beide.
48. Na het ongeluk werd de vrouw per ambulance naar het/*de ziekenhuis gebracht.
    Na de wedstrijd werd de vrouw met een taxi naar het/*de ziekenhuis gebracht.

Determiner-adjective-noun, common gender

49. Toen de kinderen op straat speelden schopten ze de/*het rode bal tegen de stoeprand.
    Toen de studenten naar huis liepen vonden ze de/*het rode bal aan de kant van de weg.
50. Tijdens een wandeling door Den Haag verdwaalde Sonja en kwam terecht in de/*het rijke buurt van de stad.
    Vanuit het bosrijke gebied vertrokken de wandelaars in de richting van de/*het rijke buurt van de stad.
51. Bij de ingang van het restaurant hield de portier de/*het grote deur open voor de klanten.
    Een foto van de familie stond op het kastje vlak naast de/*het grote deur van de slaapkamer.
52. Carolien werd geopereerd door de/*het knappe dokter van orthopedie.
    In de deuropening stond de/*het knappe dokter van orthopedie.
53. Sasha sliep ontzettend slecht nadat ze in de bioscoop de/*het enge film had gezien.
    Sasha moest lachen van de zenuwen nadat ze bij haar vriend de/*het enge film had gezien.
54. Nadat Tom zijn biertje opgedronken had, gooide hij de/*het lege fles zomaar in de prullenbak.
    Nadat Tom de barbecue had aangestoken, gooide hij de/*het lege fles benzine in de prullenbak.
55. Het toilet bevindt zich aan het einde van de/*het lange gang van het kasteel.
    Sabrina legde de boodschappen midden in de/*het lange gang van het kasteel.
56. Diana bedankte haar kraamverzorgster voor de/*het goede hulp die ze kreeg tijdens haar zwangerschap.
    Diana vertelde haar collega’s over de/*het goede hulp die ze kreeg tijdens haar zwangerschap.
57. De student betaalde een te hoge huur voor de/*het kleine kamer boven het café.
    De buurman zag een prachtige plant in de/*het kleine kamer boven het café.
58. Het nieuwe huis wordt gebouwd aan de/*het andere kant van het kleine eiland.
    De eigenaar van het grote huis heeft de/*het kant van het eiland nog nooit gezien.
59. Peter zet het prachtige servies in de/*het oude kast van zijn opa.

Peter zoekt naar belangrijke papieren in de/*het oude kast van zijn opa.

60. Marlies haar artikel kan in januari worden gelezen in de/*het nieuwe krant van Amsterdam.
Marlies doet een onderzoek naar de betrouwbaarheid van de/*het nieuwe krant van Amsterdam.
61. De indiaan voerde een heftige discussie met de/*het witte man die zijn land wilde kopen.
De schrijfster beschreef laatst voor het eerst de/*het witte man die zoveel had betekend voor de indianenstam.
62. Na de verkiezingen werd Wouter Bos benoemd tot de/*het nieuwe minister van Financiën.
Iedereen vraagt zich af wie zich binnenkort de/*het nieuwe minister van Financiën mag noemen.
63. Vera gaat vanavond naar een optreden van de/*het goede muzikant André Rieu.
Vera hoorde laatst dat ze familie is van de goede muzikant André Rieu.
64. De kok besloot de aardappels in de/*het diepe pan te koken.
De vrouw besloot de brandnetels in de/*het diepe pan te koken.
65. Peter is geboren in een klein plaatsje en verdwaalt vaak in de/*het grote stad waar hij nu woont.
Peter is een fanatieke hordeloper en traint vaak in de/*het grote stad waar hij nu woont.
66. Tijdens het optreden werd het publiek verrast door de/*het zuivere stem van een zangeres.
Tijdens de vlucht werden de passagiers verrast door de/*het zuivere stem van een zangeres.
67. De mevrouw zette al het eten op de/*het grote tafel in het restaurant.
De mevrouw bracht de dozen naar de/*het grote tafel in het restaurant.
68. Sandra was te laat voor de vergadering, omdat ze in haar agenda de/*het foute tijd had opgeschreven.
Sandra begreep niets van de Franse brigadier en had daardoor de/*het foute tijd in haar agenda gezet.
69. In het bejaardentehuis wandelt de/*het oude vrouw door de tuin.
Op het politiebureau ziet Pim de/*het oude vrouw koffie drinken.
70. Het verkeer raast elke ochtend over de/*het lange weg die de mensen naar Rotterdam brengt.
De advocaat kijkt verbaasd naar de/*het lange weg die langs zijn huis gebouwd wordt.
71. Alle Kamerleden gingen akkoord met de/*het nieuwe wet tegen kindermishandeling.
Alle leerlingen waren enthousiast over de/*het nieuwe wet tegen kindermishandeling.
72. De Fransman eet altijd stokbrood en drinkt daarbij de/*het goede wijn uit Frankrijk.
De Groninger eet altijd boerenkool en neemt daarbij de goede wijn uit Frankrijk.

Determiner-adjective-noun, neuter gender

73. Bianca leest vol spanning het/*de nieuwe boek van haar favoriete schrijfster.
Bianca zoekt al weken naar het/*de nieuwe boek van haar favoriete schrijfster.
74. Van de katachtigen is de tijger het/*de grootste dier dat er bestaat.
De promovendus bestudeert het/*de grootste dier dat er bestaat.
75. Sandra is geboren en getogen in het/*de kleine dorp Walem in Zuid-Limburg.
Sandra gaat graag met haar moeder naar het/*de kleine dorp Walem in Zuid-Limburg.
76. De vrienden van de jarige verheugden zich al lang op de het/*de grote feest op het strand.
Vlak voordat de vrouw een ongeluk kreeg was ze op het/*de grote feest aan het strand.
77. De technicus kwam er pas na het concert achter wat het/*de vreemde geluid had veroorzaakt.
De zuster van het verzorgingstehuis kwam al snel het/*de vreemde geluid op het spoor.
78. De koeien renden het weiland in en aten het/*de hoge gras langs de sloot.
De jongens speelden met autootjes in het/*de hoge gras langs de sloot.
79. Omdat ze zich schaamde toen de foto werd genomen is Liesbeth makkelijk te herkennen aan het/*de rode hoofd dat boven de groep uitsteekt.
Het vriendje van Liesbeth is voor het eerst bij de familiedag, maar hij is makkelijk te herkennen

aan het/*de rode hoofd dat boven de groep uitsteekt.

80. Toen hij zijn ouders kwijt was geraakt schreeuwde het/*de kleine kind totdat iemand hem optilde.
    De automonteur liep graag langs water met het/*de kleine kind van zijn broer.
81. In de woning trof de rechercheur het/*de lichaam aan van de wapenhandelaar.
    In de tuin trof de kleuter het/*de dode lichaam aan van een lieveheersbeestje.
82. Het professionele koor zong een nieuwe versie van het/*de prachtige lied over de liefde.
    Het onzekere meisje gaf haar familie een voorproefje van het/*de prachtige lied over de liefde.
83. Om het vlees in kleine stukjes te snijden gebruikte Marco het/*de nieuwe mes van de slager.
    Om zijn vervelende vrienden te pesten gebruikte Marco het/*de nieuwe mes van de slager.
84. Mark wil erg graag zijn vriendin ten huwelijk vragen, maar wacht op het/*de juiste moment om dit te doen.
    Mark wil zijn nieuwe recept uitproberen, maar heeft nog niet het/*de juiste moment gevonden om dit te doen.
85. Kim krijgt een onbekende man aan de telefoon en vraagt zich af of ze het/*de juiste nummer heeft gedraaid.
    Kim heeft haar zaken vandaag niet op orde en vraagt zich af of ze het/*de juiste nummer heeft gedraaid.
86. Na de vechtpartij liep Joris nog weken rond met het/*de blauwe oog en de pijnlijke vinger.
    Na het schoolbal herkende Joris zijn vader aan het/*de blauwe oog en de pijnlijke vinger.
87. Tijdens haar eerste rijles op de manege reed Leonie vol trots op het/*de mooie paard dat ze zelf had uitgekozen.
    Tijdens haar eerste vakantie was Leonie dolgelukkig met het/*de mooie paard dat ze zelf had uitgekozen.
88. Speciaal voor de trouwerij trok Jeroen het/*de nette pak van zijn broer aan.
    Het duurde uren voordat Jeroen het/*de nette pak van zijn broer had aangetrokken.
89. Tijdens het vouwen van de envelop sneed het meisje zich aan het/*de scherpe papier en begon te huilen.
    Tijdens het spelen sneed het meisje zich per ongeluk aan het/*de scherpte papier van een envelop.
90. Volgens de minister vormt overgewicht bij kinderen het/*de grote probleem in de samenleving.
    Volgens de voetballer zal zijn jaloerse vrouw het/*de grote probleem vormen dit voetbalseizoen.
91. Na het optreden kreeg de band een wild applaus van het/*de leuke publiek in de zaal.
    Na de zwemles kreeg het jongetjes de zenuwen van het/*de leuke publiek in de zaal.
92. Op 31 december eten we oliebollen tijdens het/*de laatste uur van het jaar.
    Karin en Greet kijken al weken lang uit naar het/*de laatste uur van het jaar.
93. De lerares vertelde de kinderen het/*de mooie verhaal van roodkapje.
    De lerares was niet bekend met het/*de mooie verhaal van roodkapje.
94. Door mensen met respect te behandelen wil Marga haar kinderen het/*de goede voorbeeld geven.
    Door iedere avond te gaan hardlopen wil Marga proberen het/*de goede voorbeeld te geven.
95. De zakenman en zijn minnares hebben een hotel in de Ardennen geboekt voor het/*de lange weekend van Pinksteren.
    De minnares van de zakenman is compleet verbaasd als ze het/*de lange weekend in een luxe hotel mag doorbrengen.
96. Tijdens de finale van zijn spellingwedstrijd spelt Tom het/*de woord foutloos
    Tijdens zijn vakantie in Gelderland leert Tom het/*de lastige woord ‘aubergine’ te spellen.
