# Supplementary material for: An ERP study on L2 syntax processing: When do learners fail?
Source: Front Psychol. 2014 Sep 25;5:1072. doi: 10.3389/fpsyg.2014.01072 (PMC4174886; doi:10.3389/fpsyg.2014.01072)
Supplement: Supplementary file 2 [file DataSheet2.PDF]

Supplementary Material

An ERP study on L2 syntax processing: When do learners fail?

Nienke Meulman[1,*], Laurie A. Stowe[1], Simone A. Sprenger[1], Moniek Bresser[2], Monika S. Schmid[1,3]

[1]Center for Language and Cognition, University of Groningen, Groningen, The Netherlands
[2]Research School of Behavioral and Cognitive Neurosciences, University of Groningen, Groningen, The Netherlands
[3]Department of Language and Linguistics, University of Essex, Colchester, United Kingdom

***Correspondence:**
Nienke Meulman
Center for Language and Cognition
University of Groningen
Oude Kijk in 't Jatstraat 26
P.O. Box 716, 9700 AS
Groningen, The Netherlands
n.meulman@rug.nl

Data sheet 2: Results of the omnibus ANOVA

The results of the omnibus ANOVAs conducted on the grand mean ERPs are shown below. Results are reported for the 300–500 ms and the 600–1200 ms window, for the non-finite verb (VERB) and gender (GEN) condition. The analyses included the factors C=Correctness, G=Group, M=Modality, A=Anterior-Posterior and H=Hemisphere. The Greenhouse-Geisser correction was applied for violations of the sphericity assumption.

Significance codes: '***' = 0.001, '**' = 0.01, '*' = 0.05, '(*)' = 0.1.

Effect	VERB (300–500 ms)	VERB (600–1200 ms)	GEN (300–500 ms)	GEN (600–1200)
Lateral ANOVA:				
C	F(1,36) = 5.16, p = .029*	F(1,36) = 33.29, p < .001***	F(1,36) = 0.76, p = .388	F(1,36) = 11.91, p = .001**
G × C	F(1,36) = 3.65, p = .064(*)	F(1,36) = 0.00, p = .949	F(1,36) = 0.49, p = .488	F(1,36) = 13.12, p = .001**
C × M	F(1,36) = 0.97, p = .331	F(1,36) = 0.44, p = .510	F(1,36) = 0.06, p = .811	F(1,36) = 0.20, p = .657
C × A	F(3,108) = 6.02, p = .011*	F(3,108) = 17.95, p < .001***	F(3,108) = 0.95, p = .366	F(3,108) = 16.16, p < .001***
C × H	F(1,36) = 2.46, p = .126	F(1,36) = 1.08, p = .306	F(1,36) = 1.44, p = .239	F(1,36) = 6.50, p = .015*
G × C × M	F(1,36) = 1.39, p = .246	F(1,36) = 0.25, p = .618	F(1,36) = 0.16, p = .692	F(1,36) = 0.75, p = .394
G × C × A	F(3,108) = 0.36, p = .617	F(3,108) = 5.95, p = .008**	F(3,108) = 0.45, p = .573	F(3,108) = 20.17, p < .001***
G × C × H	F(1,36) = 0.18, p = .672	F(1,36) = 1.07, p = .308	F(1,36) = 2.04, p = .162	F(1,36) = 2.24, p = .144
C × M × A	F(3,108) = 0.90, p = .383	F(3,108) = 1.46, p = .241	F(3,108) = 3.90, p = .039*	F(3,108) = 7.31, p = .002**
C × M × H	F(1,36) = 1.09, p = .304	F(1,36) = 0.08, p = .783	F(1,36) = 1.01, p = .321	F(1,36) = 0.51, p = .481
C × A × H	F(3,108) = 2.31, p = .093(*)	F(3,108) = 2.66, p = .064(*)	F(3,108) = 1.41, p = .250	F(3,108) = 1.48, p = .231
G × C × M × A	F(3,108) = 0.30, p = .675	F(3,108) = 1.45, p = .243	F(3,108) = 2.10, p = .145	F(3,108) = 1.05, p = .349
G × C × M × H	F(1,36) = 0.01, p = .905	F(1,36) = 0.89, p = .353	F(1,36) = 5.24, p = .028*	F(1,36) = 0.35, p = .558
G × C × A × H	F(3,108) = 2.13, p = .113	F(3,108) = 2.73, p = .059(*)	F(3,108) = 1.55, p = .217	F(3,108) = 0.84, p = .452
C × M × A × H	F(3,108) = 1.12, p = .344	F(3,108) = 1.12, p = .344	F(3,108) = 0.12, p = .875	F(3,108) = 0.31, p = .752
G × C × M × A × H	F(3,108) = 1.61, p = .191	F(3,108) = 1.78, p = .155	F(3,108) = 1.06, p = .349	F(3,108) = 1.35, p = .265
Medial ANOVA:				
C	F(1,36) = 9.22, p = .004**	F(1,36) = 52.19, p < .001***	F(1,36) = 0.08, p = .775	F(1,36) = 17.23, p < .001***
G × C	F(1,36) = 0.81, p = .374	F(1,36) = 0.31, p = .580	F(1,36) = 4.30, p = .045*	F(1,36) = 21.72, p < .001***
C × M	F(1,36) = 0.27, p = .605	F(1,36) = 0.07, p = .793	F(1,36) = 0.26, p = .615	F(1,36) = 1.41, p = .244
C × A	F(1,36) = 3.33, p = .076(*)	F(1,36) = 22.93, p < .001***	F(1,36) = 0.79, p = .381	F(1,36) = 24.74, p < .001***
G × C × M	F(1,36) = 3.56, p = .067(*)	F(1,36) = 1.48, p = .231	F(1,36) = 0.10, p = .751	F(1,36) = 1.27, p = .266
G × C × A	F(1,36) = 0.06, p = .807	F(1,36) = 1.73, p = .197	F(1,36) = 1.32, p = .258	F(1,36) = 11.24, p = .002**
C × M × A	F(1,36) = 0.06, p = .801	F(1,36) = 0.02, p = .879	F(1,36) = 0.62, p = .438	F(1,36) = 1.39, p = .246
G × C × M × A	F(1,36) = 0.13, p = .725	F(1,36) = 0.39, p = .535	F(1,36) = 0.60, p = .443	F(1,36) = 1.94, p = .172
